# Supplementary material for: Nopol-Based Quinoline Derivatives as Antiplasmodial Agents
Source: Molecules. 2021 Feb 14;26(4):1008. doi: 10.3390/molecules26041008 (PMC7917639; doi:10.3390/molecules26041008)
Supplement: Supplementary file 1 [file molecules-26-01008-s001.pdf]

Nopol-Based Quinoline Derivatives as Antiplasmodial Agents

Rogers J. Nyamwihura, Huaisheng Zhang, Jasmine T. Collins, Olamide Crown, Ifedayo Victor Ogungbe*

Department of Chemistry, Physics, and Atmospheric Sciences, Jackson State University, Jackson, MS, 39217, USA

* Correspondence: ifedayo.v.ogungbe@jsums.edu; Tel.:+1-601-979-3719

Supplementary NMR and High-Resolution Mass Spectrometry (HRMS) Data

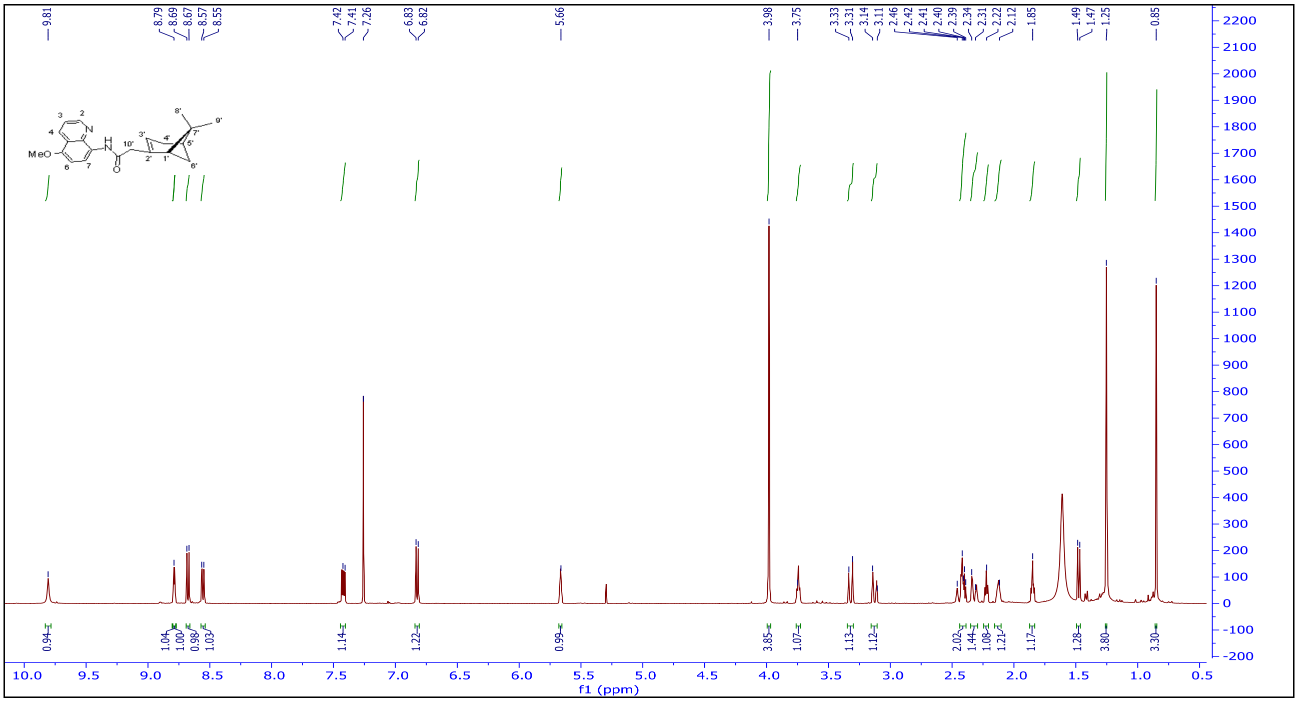

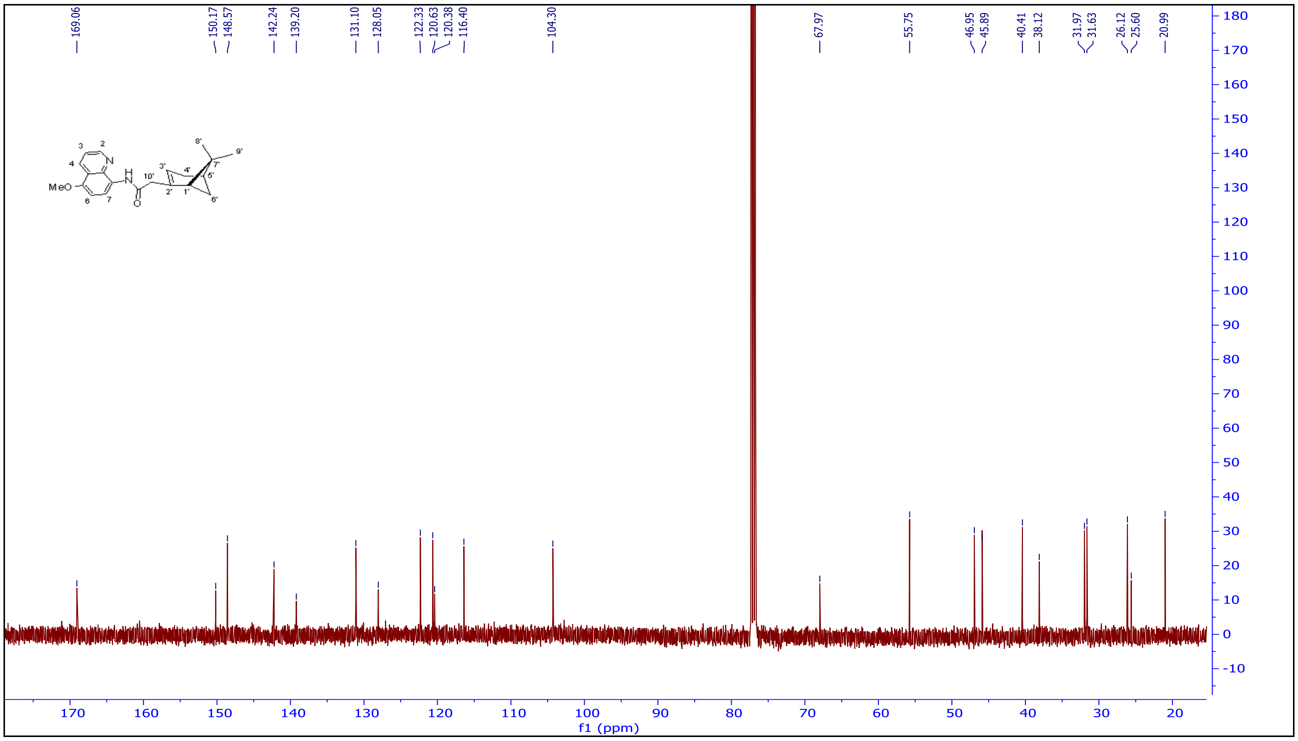

Figure 1.1a: (Top) ^{1}H-NMR and (Bottom) ^{13}C-NMR of 2-(6,6-dimethylbicyclo [3.1.1] hept-2-en-2-yl)-N-(5-methoxyquinolin-8-yl) acetamide (**1**) in $CDCl_3$.

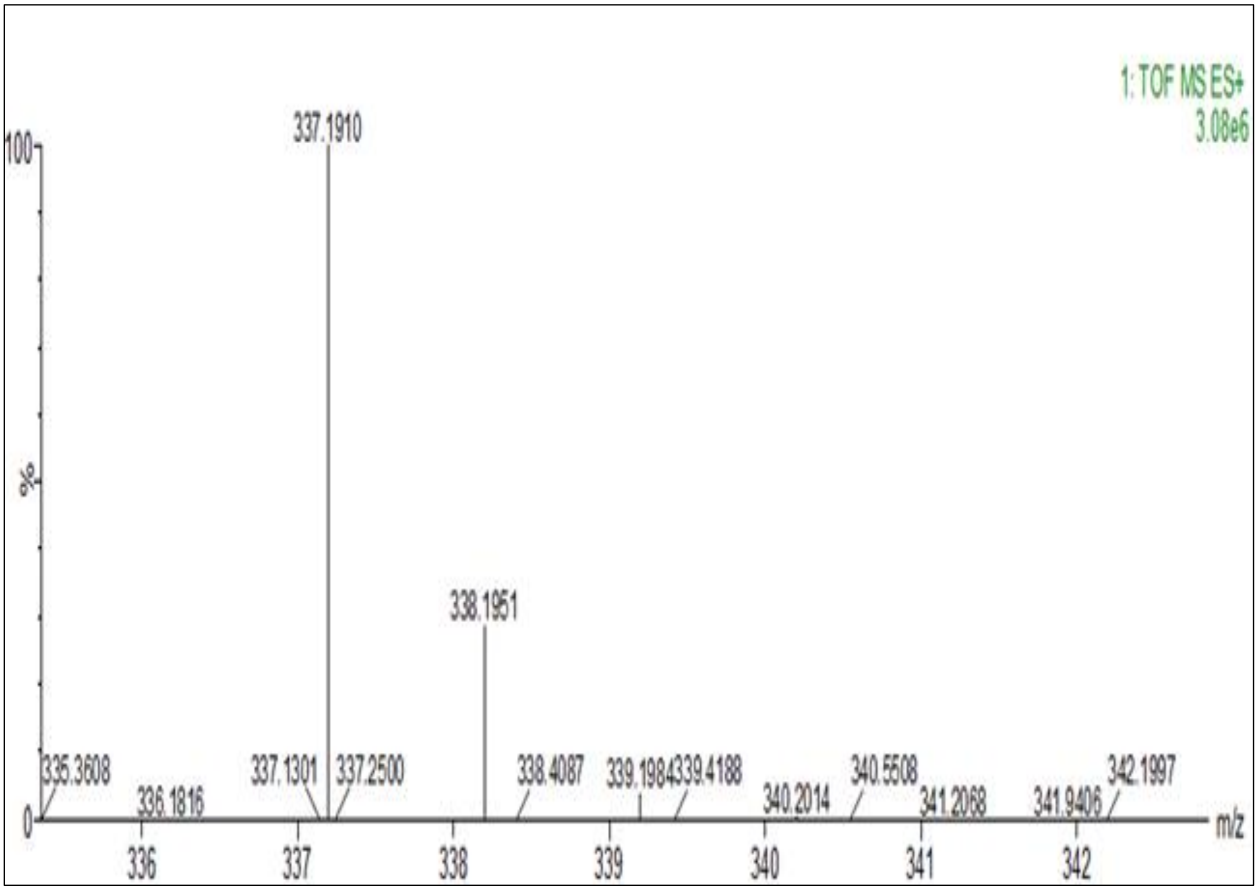

Figure 1.1b: HRMS: $[M+H]^+$: 337.1910 m/z for 2-(6,6-dimethylbicyclo [3.1.1] hept-2-en-2-yl)-N-(5-methoxyquinolin-8-yl) acetamide (**1)**.

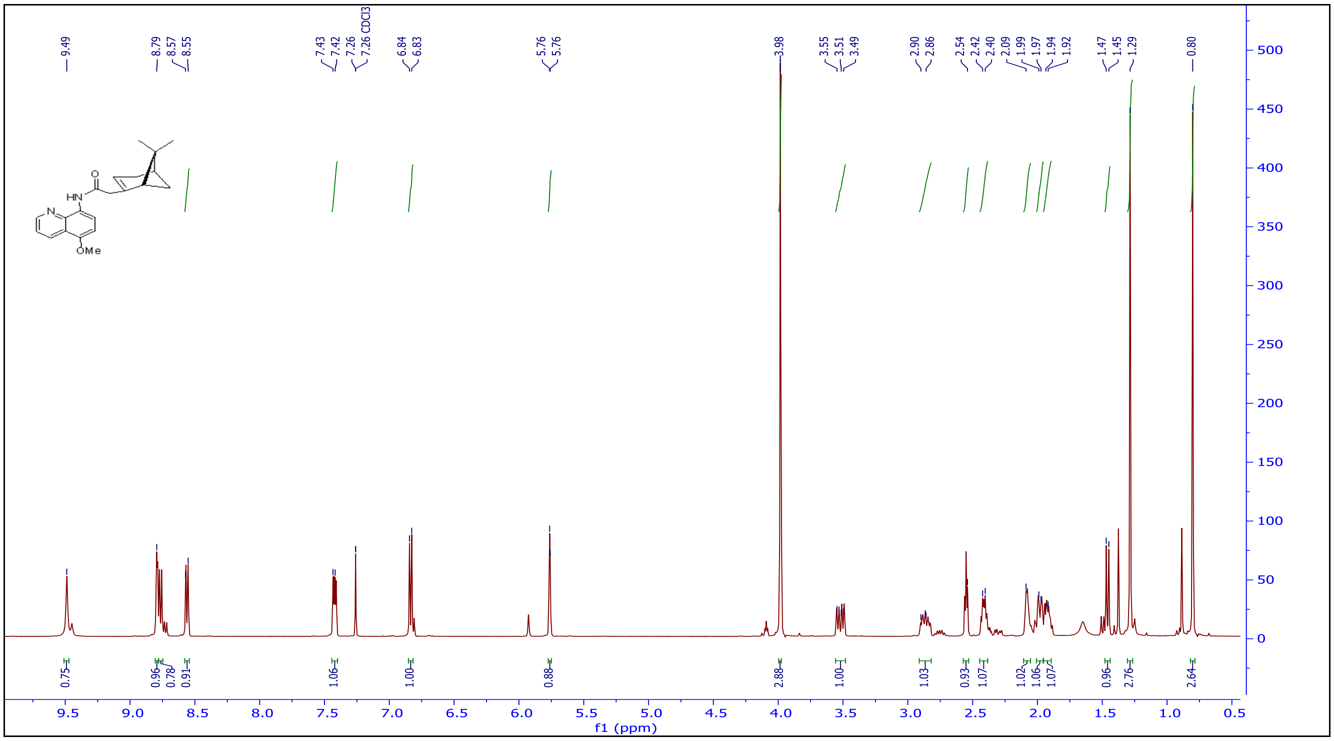

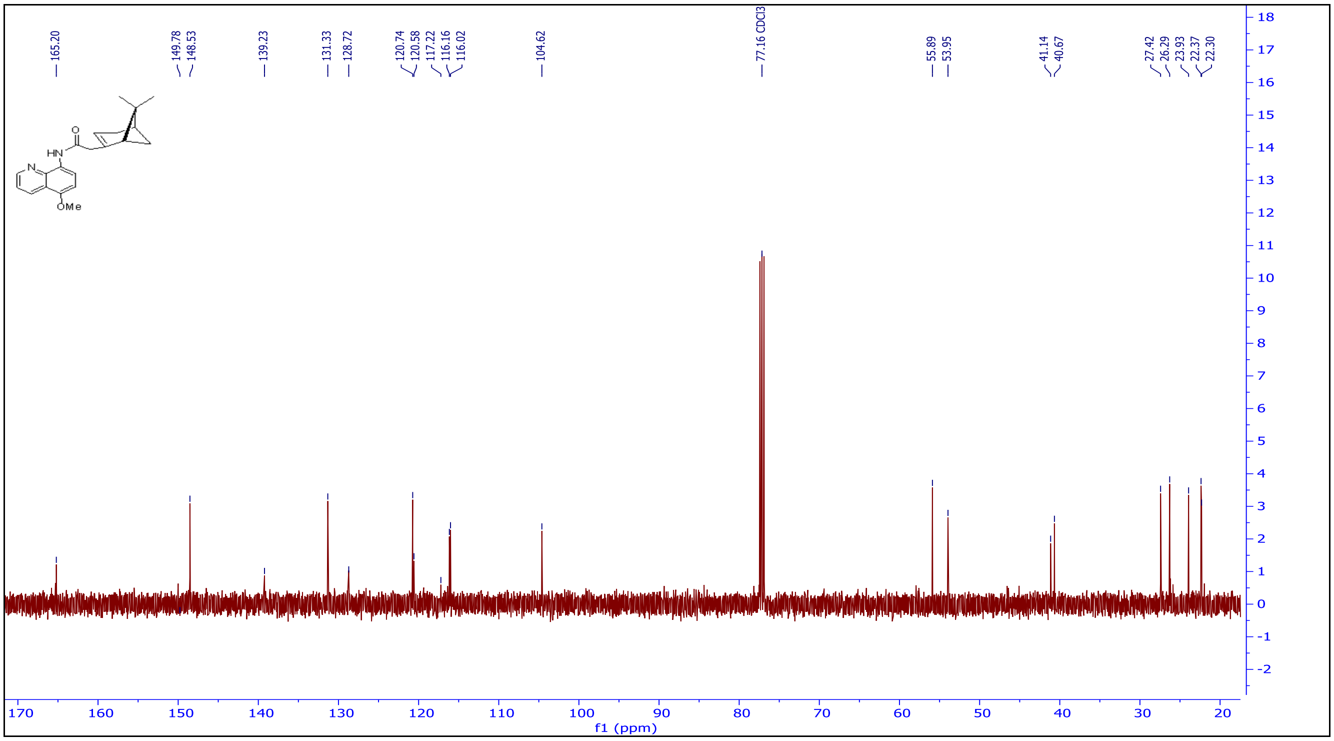

Figure 1.2a: (Top) ^{1}H-NMR and (Bottom) ^{13}C-NMR of 2-(6,6-dimethylbicyclo [3.1.1] hept-2-en-2-yl)-N-(5-methoxyquinolin-8-yl) acetamide (**2**) in $CDCl_3$.

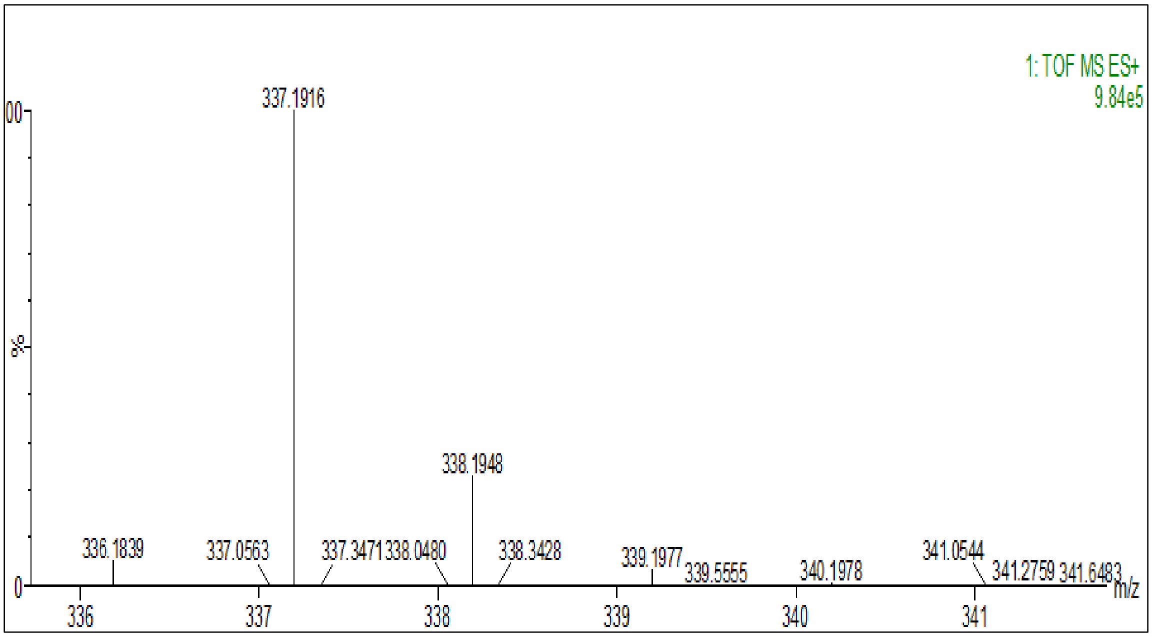

Figure 1.2b: HRMS: [M+H]$^+$: 337.1916 m/z for 2-(6, 6-dimethylbicyclo [3.1.1] hept-2-en-2-yl)-N-(5-methoxyquinolin-8-yl) acetamide (**2**).

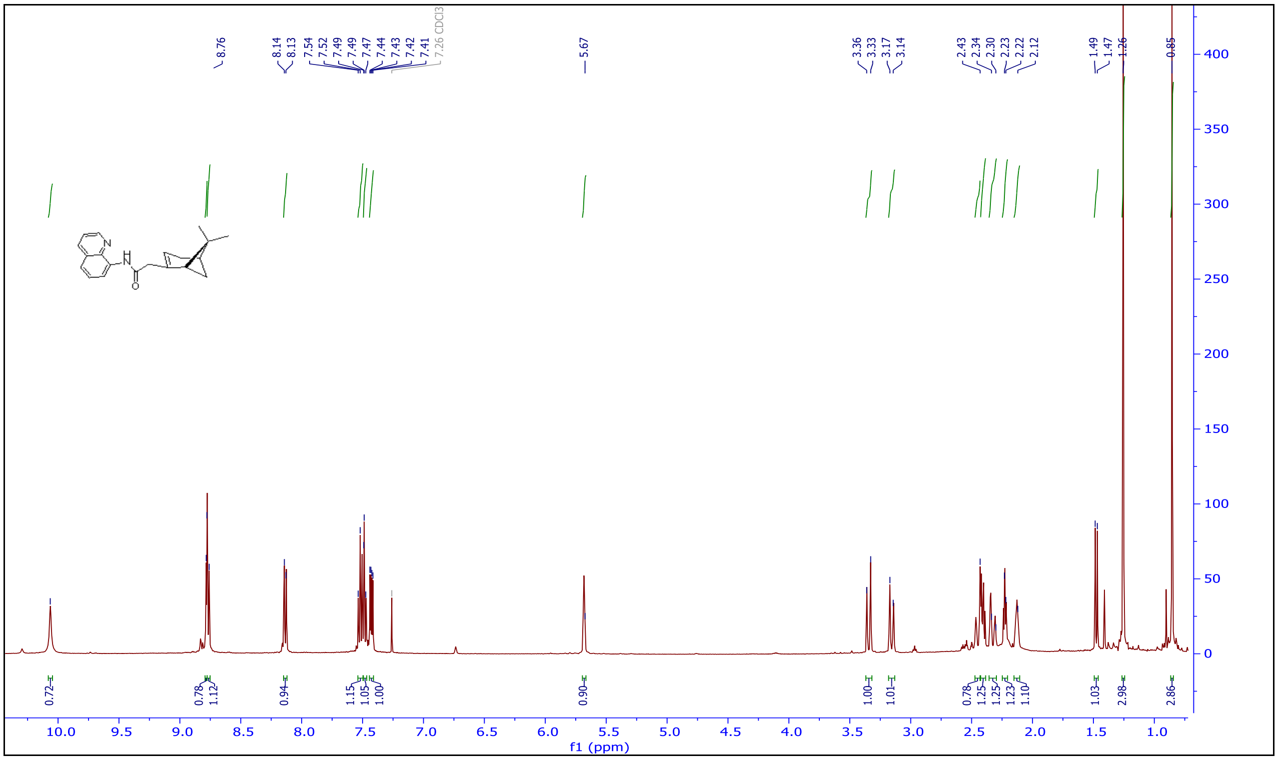

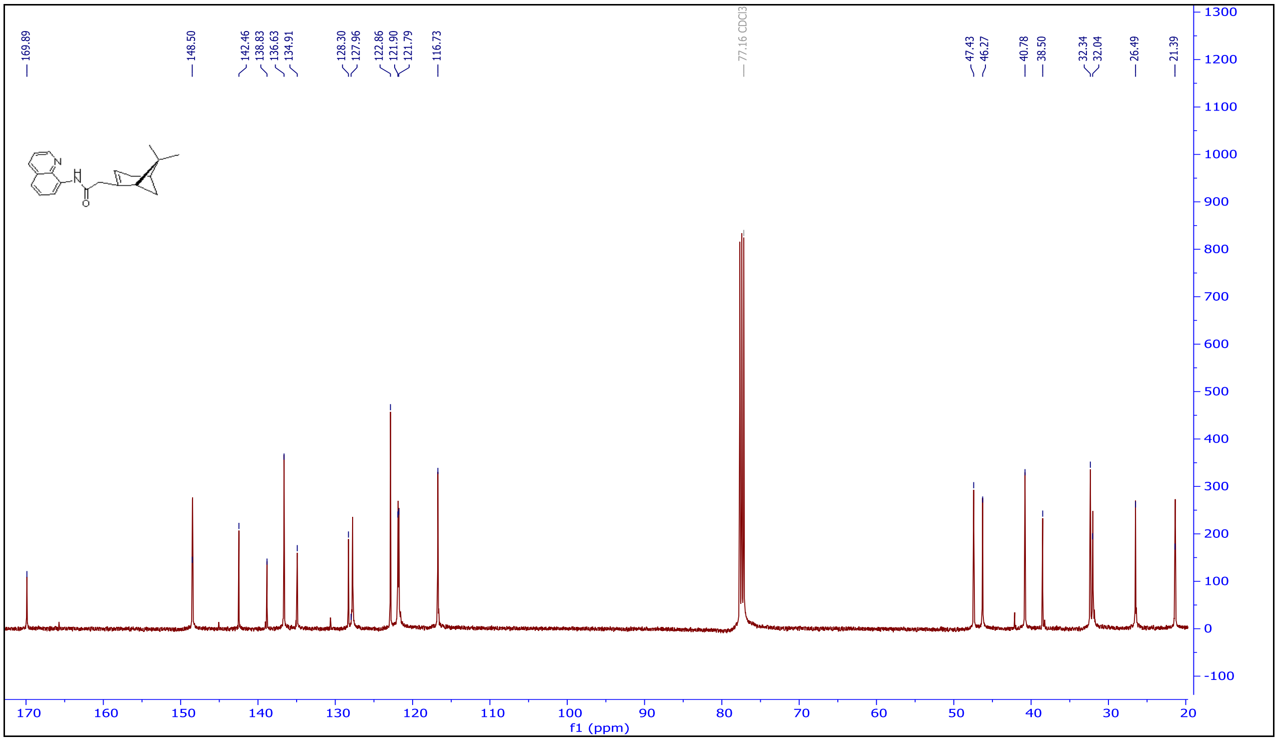

Figure1.3a: (Top) ^{1}H-NMR and (Bottom) ^{13}C-NMR of 2-(6,6-dimethylbicyclo [3.1.1] hept-2-en-2-yl)-N-(quinolin-8-yl) acetamide (**3**) in $CDCl_3$.

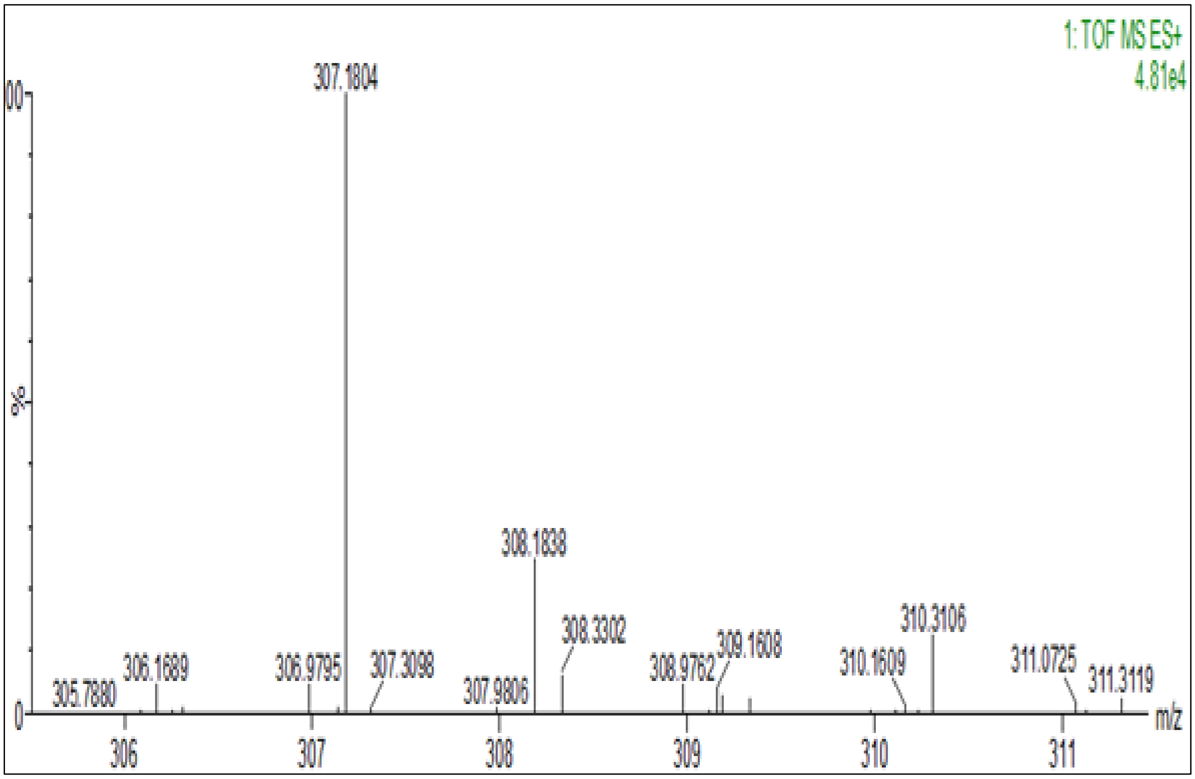

Figure 1.3b: HRMS: $[M+H]^+$: 307.1804 m/z for 2-(6,6-dimethylbicyclo [3.1.1] hept-2-en-2-yl)-N-(quinolin-8-yl) acetamide (**3**).

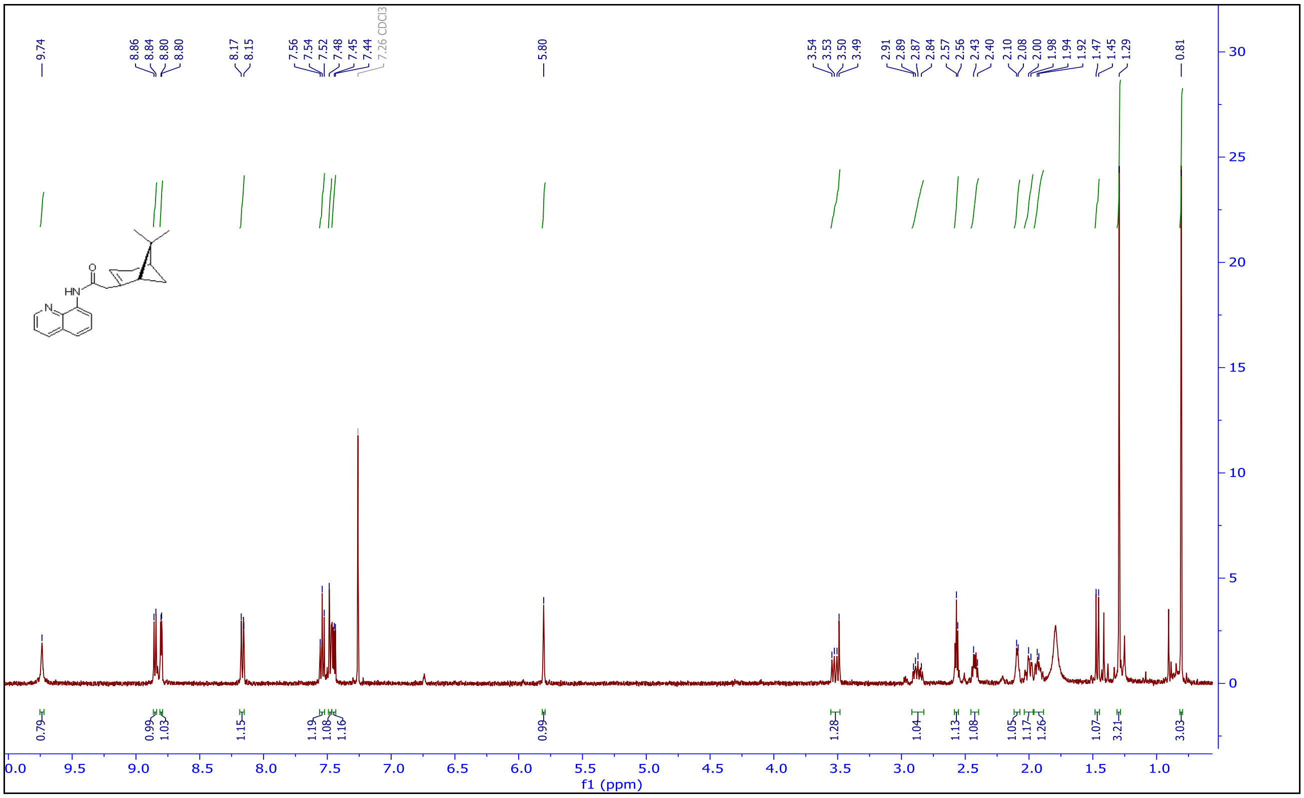

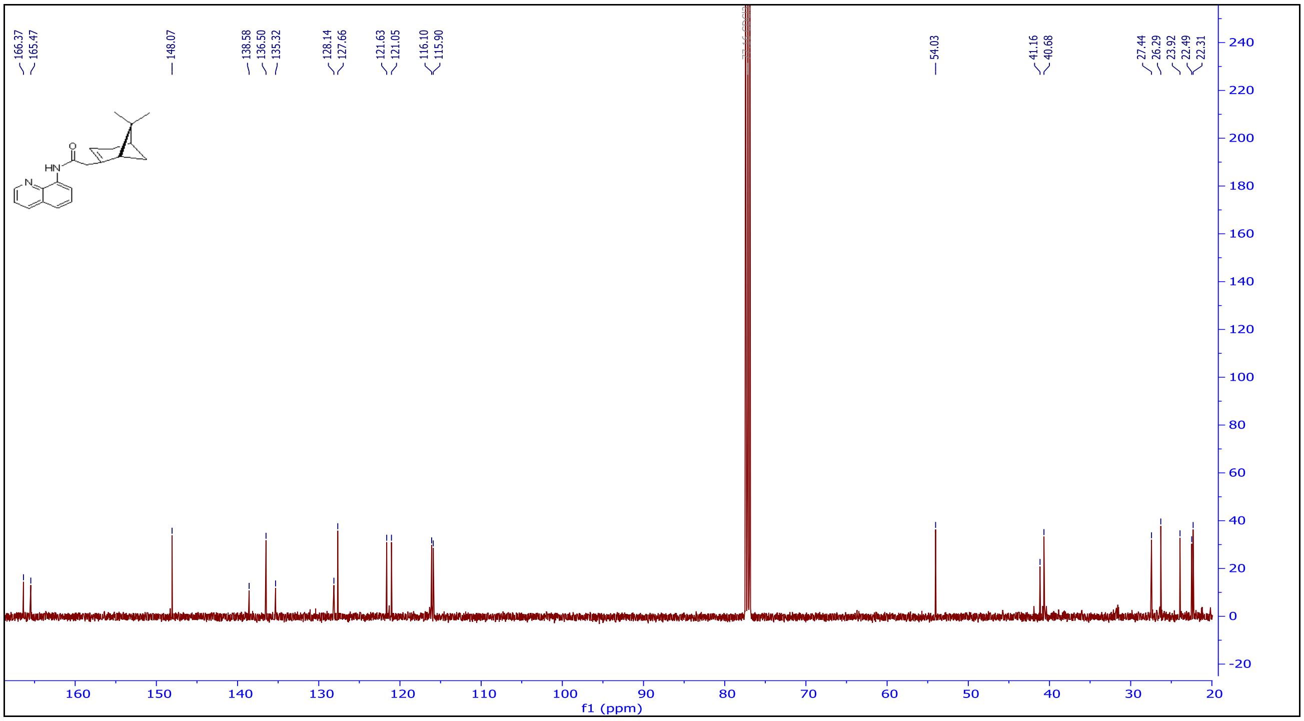

Figure 1.4a: (Top) ^{1}H-NMR and (Bottom) ^{13}C-NMR of 2-(6,6 dimethylbicyclo [3.1.1] hept-2-en-2-yl)-N-(quinolin-8-yl) acetamide (**4**) in $CDCl_3$.

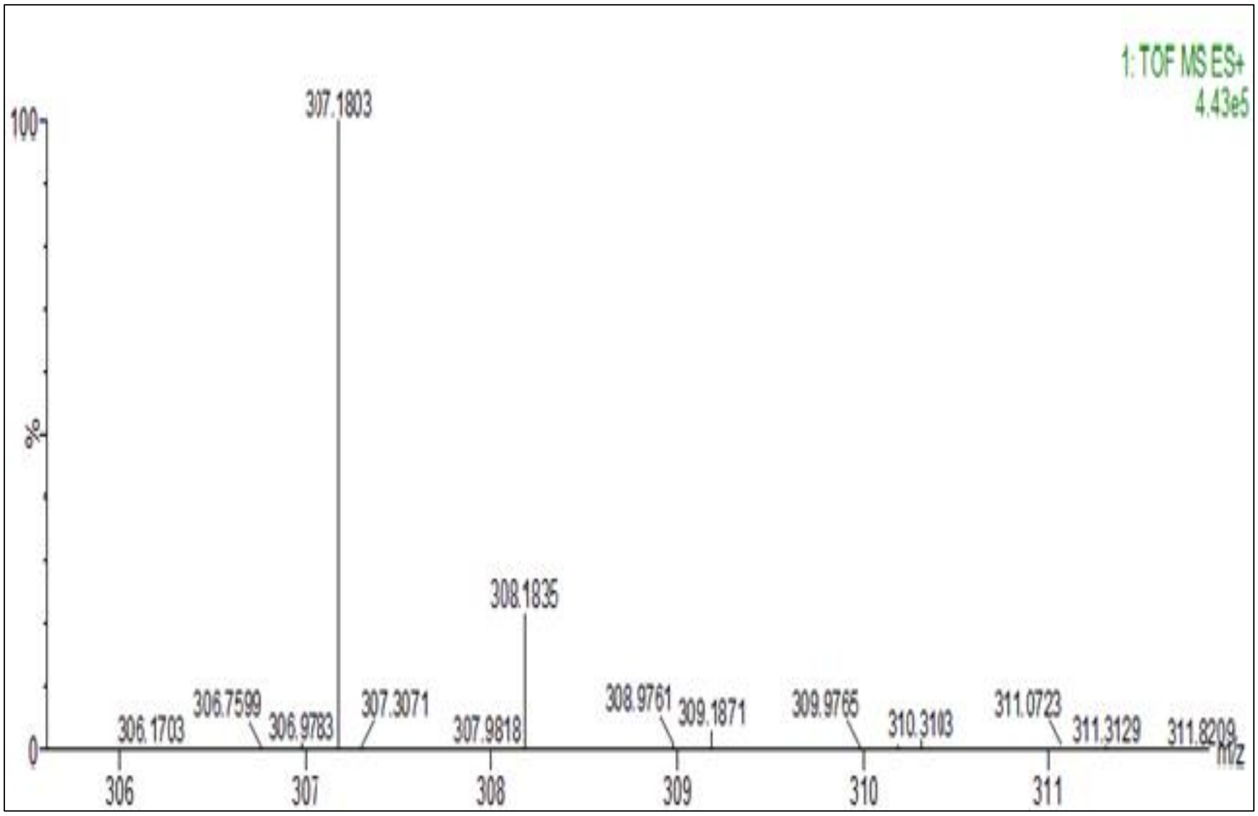

Figure 1.4b: HRMS: $[M+H]^+$: 307.1803 m/z for 2-(6,6-dimethylbicyclo [3.1.1] hept-2-en-2-yl)-N-(quinolin-8-yl) acetamide (**4**).

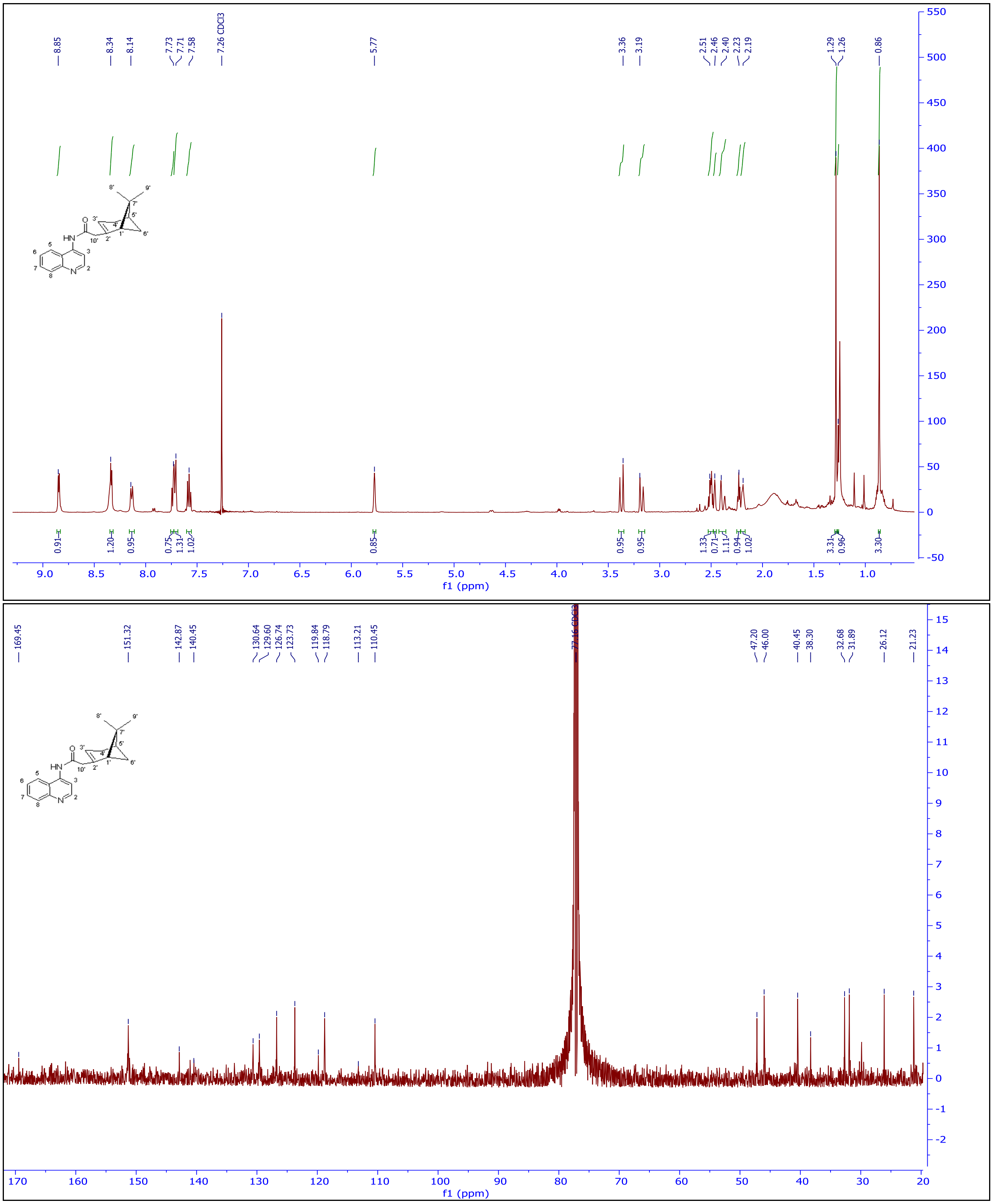

Figure 1.5a: (Top) ^{1}H-NMR and (Bottom) ^{13}C-NMR of 2-((1*S*,5*R*)-6,6-dimethylbicyclo [3.1.1] hept-2-en-2-yl)-*N*-(quinolin-4-yl) acetamide (**5**) in $CDCl_3$.

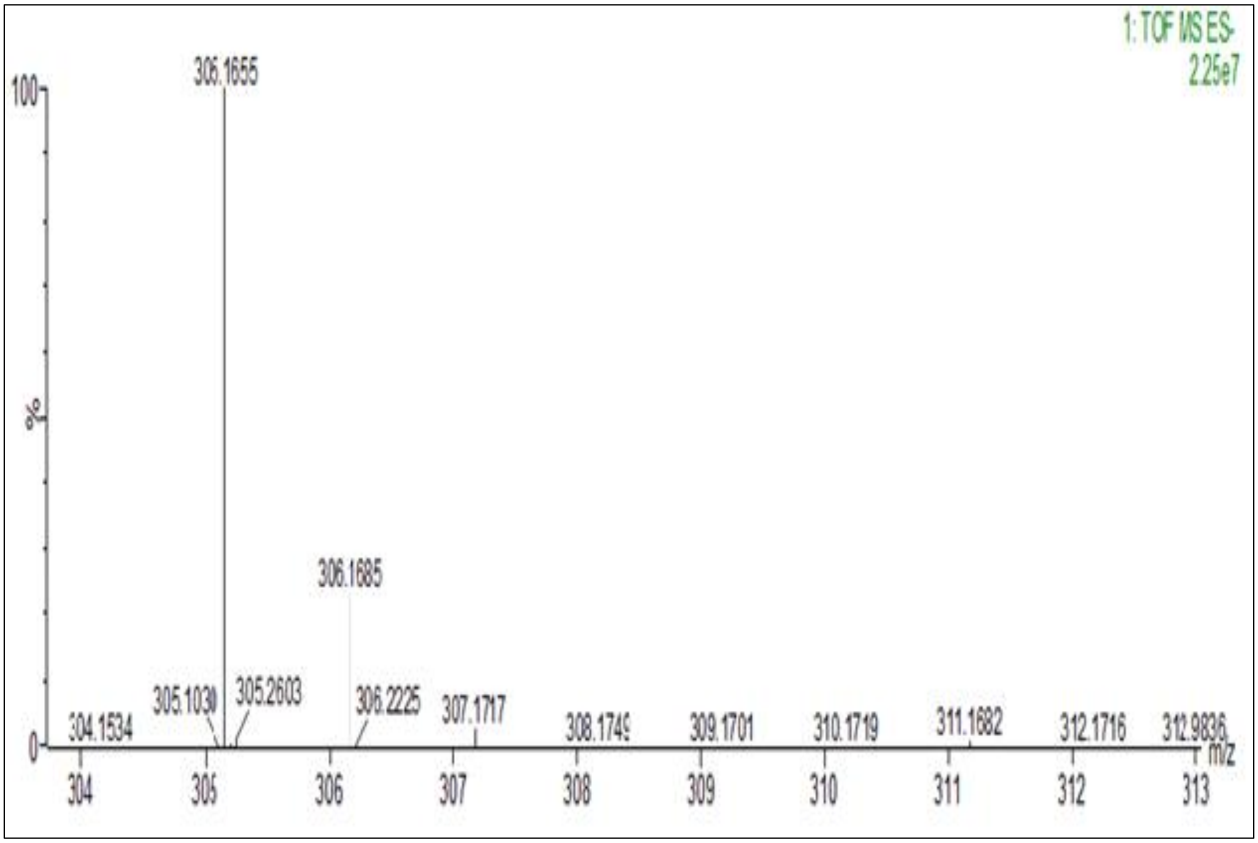

Figure 1.5b: HRMS: [M-H]$^-$: 305.1655 m/z for 2-((1*S*,5*R*)-6,6-dimethylbicyclo [3.1.1] hept-2-en-2-yl)-*N*-(quinolin-4-yl) acetamide (**5**).

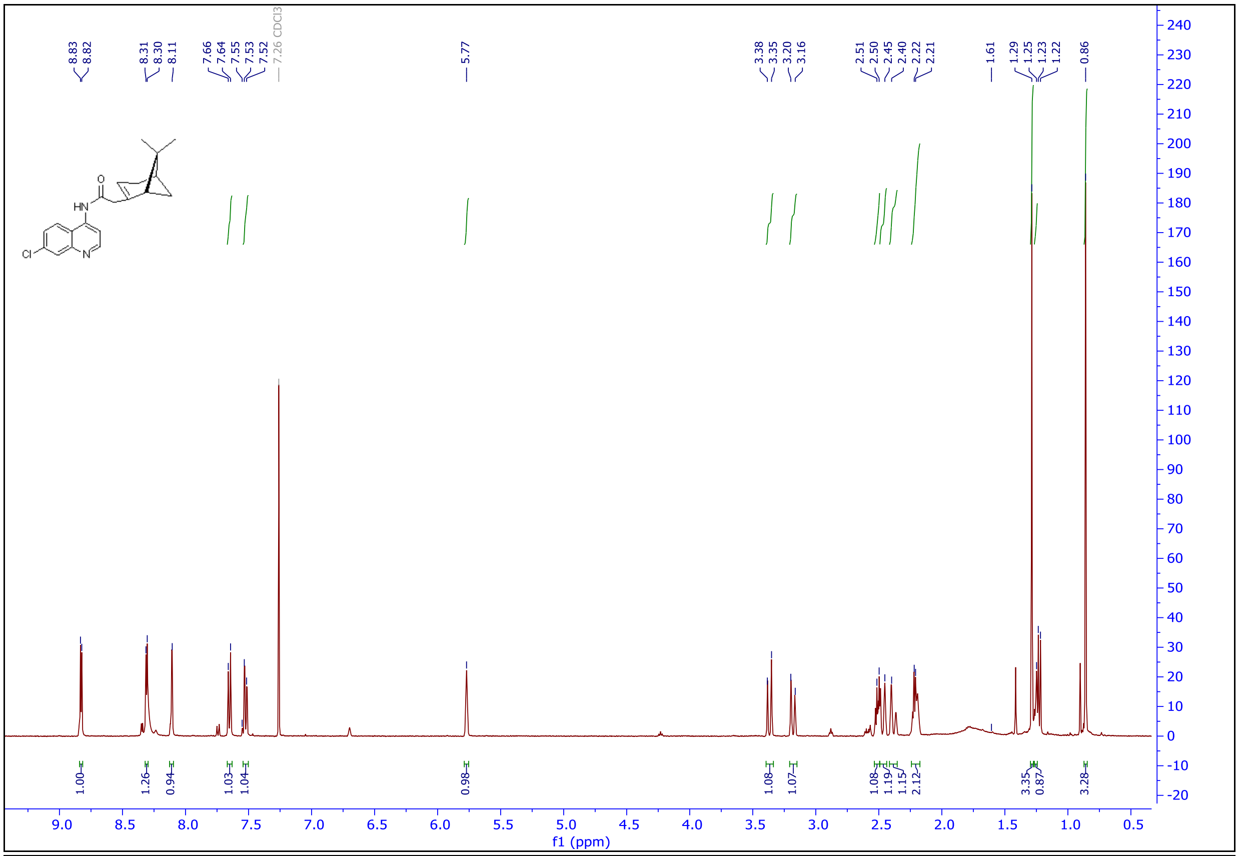

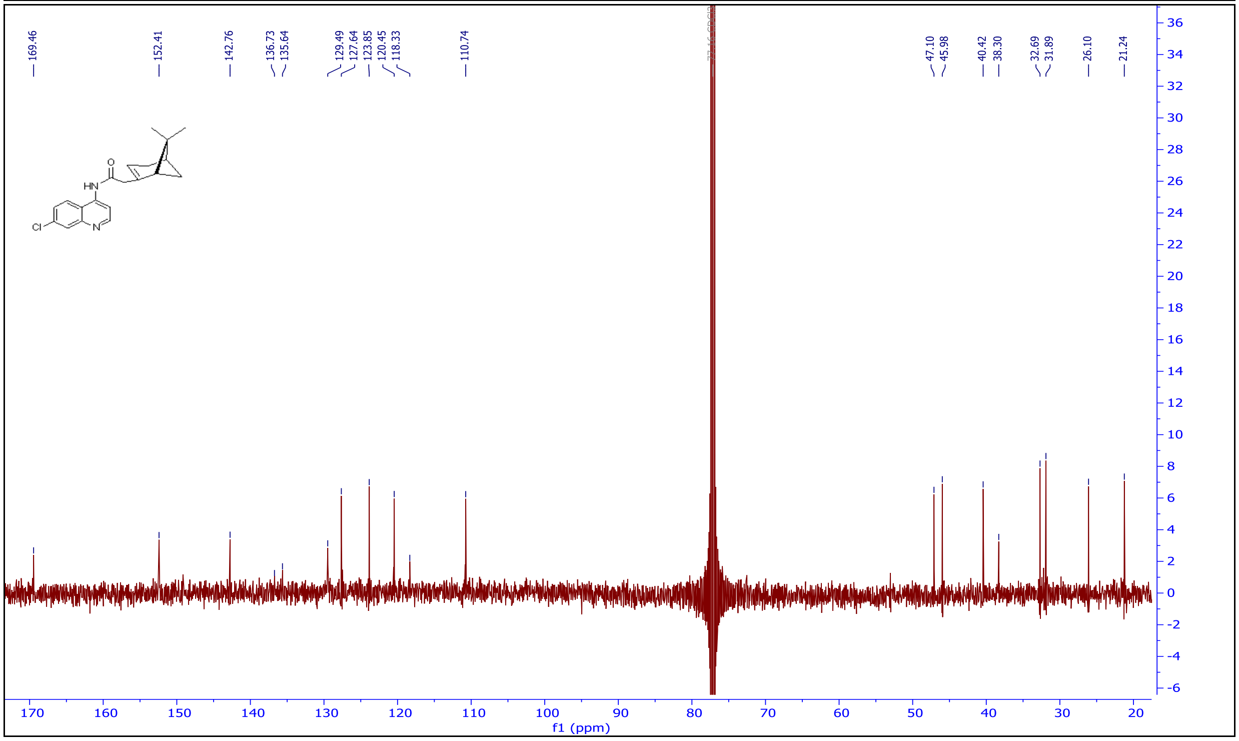

Figure 1.6a: (Top) ^{1}H-NMR and (Bottom) ^{13}C-NMR of *N*-(7-chloroquinolin-4-yl)-2-((1*S*,5*R*)-6,6-dimethylbicyclo [3.1.1] hept-2-en-2-yl) acetamide (**6**) in $CDCl_3$.

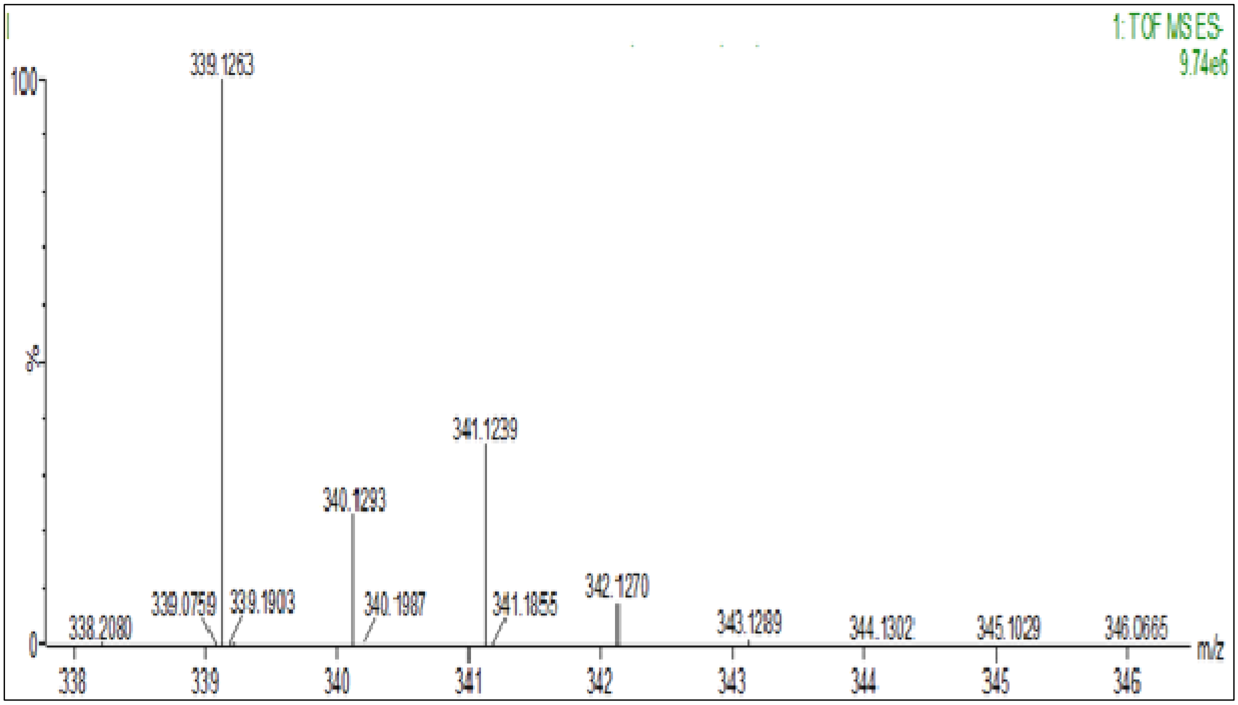

Figure 1.6b: HRMS: $[M-H]^-$: 339.1263 m/z for *N*-(7-chloroquinolin-4-yl)-2-((1*S*,5*R*)-6,6-dimethylbicyclo [3.1.1] hept-2-en-2-yl) acetamide (**6**).

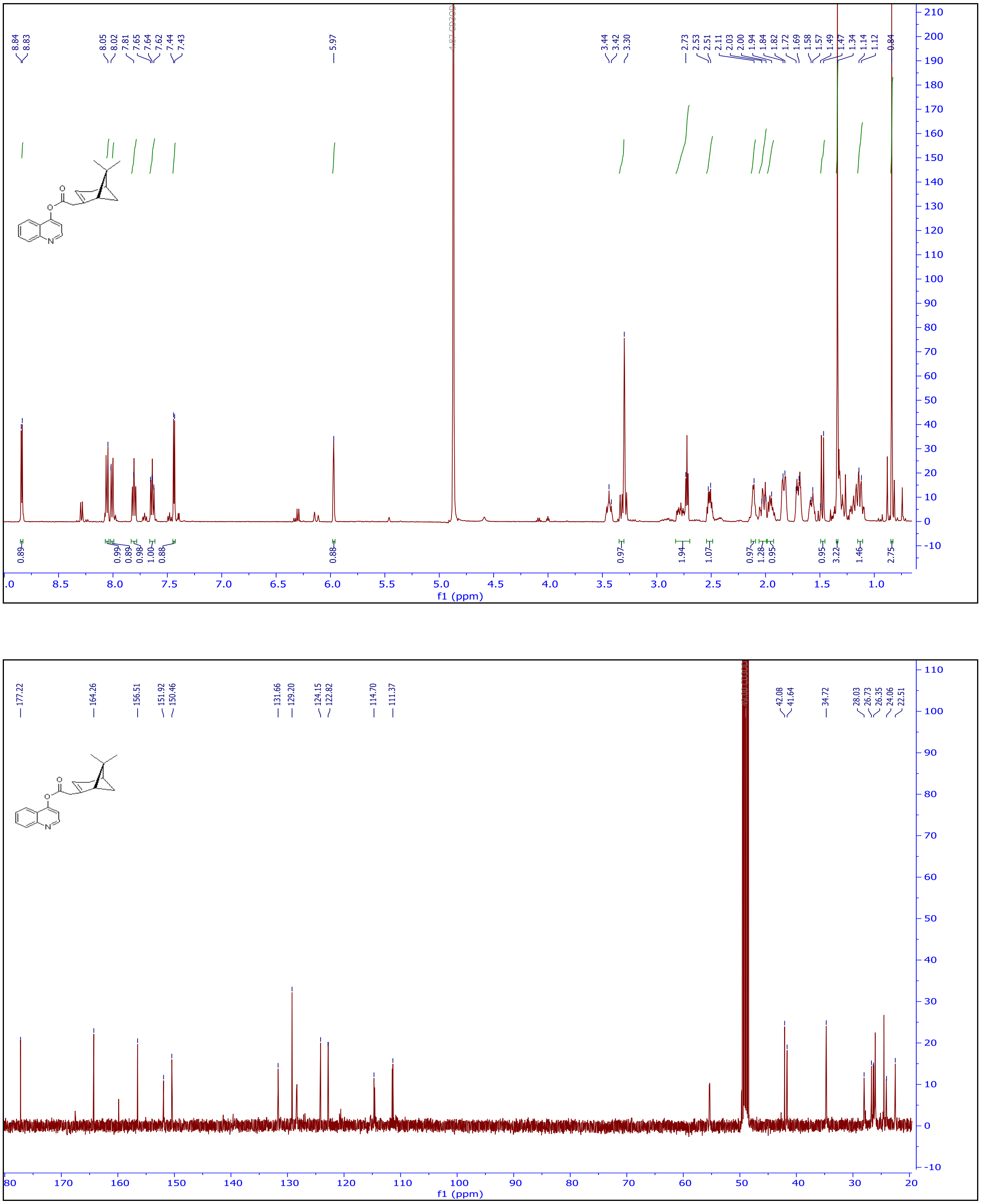

Figure 1.7a: (Top) ^{1}H-NMR and (Bottom) ^{13}C-NMR of quinolin-4-yl 2-((1*S*,5*R*)-6,6-dimethylbicyclo [3.1.1] hept-2-en-2-yl) acetate (**7**) in $CDCl_3$.

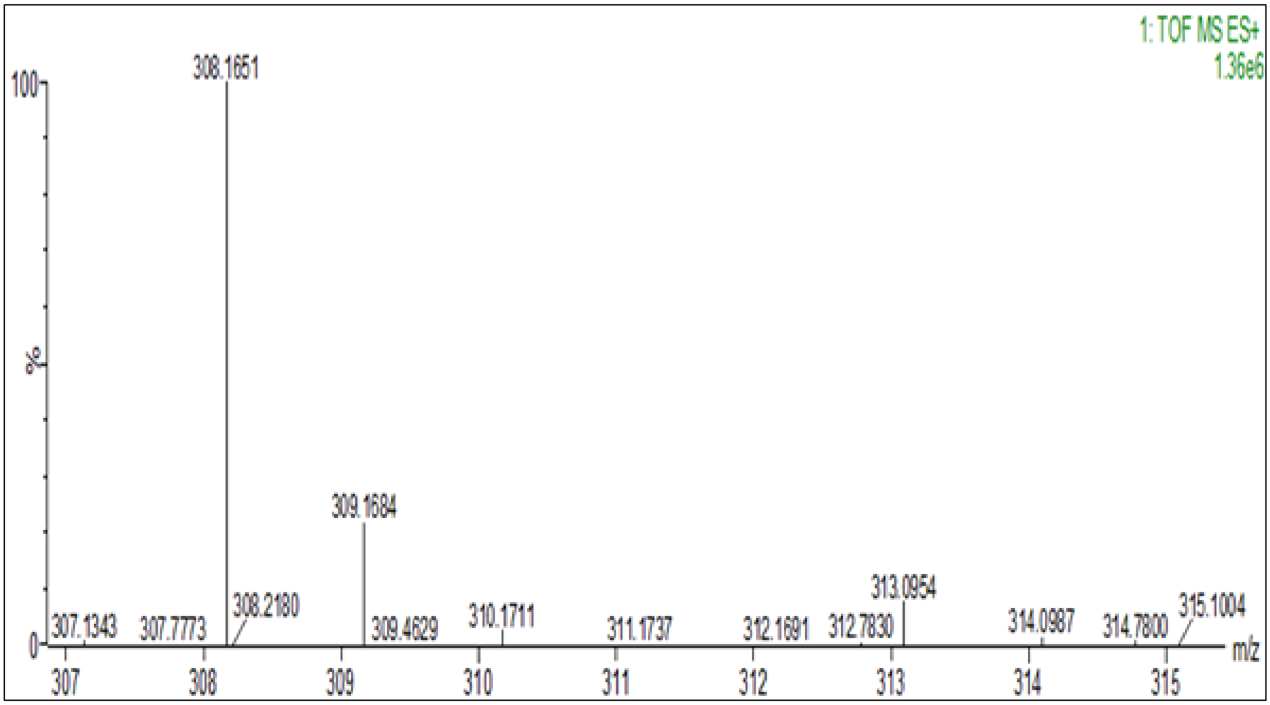

Figure 1.7b: HRMS: $[M+H]^+$: 308.1651 m/z for quinolin-4-yl 2-((1*S*,5*R*)-6,6-dimethylbicyclo [3.1.1] hept-2-en-2-yl) acetate (**7**).

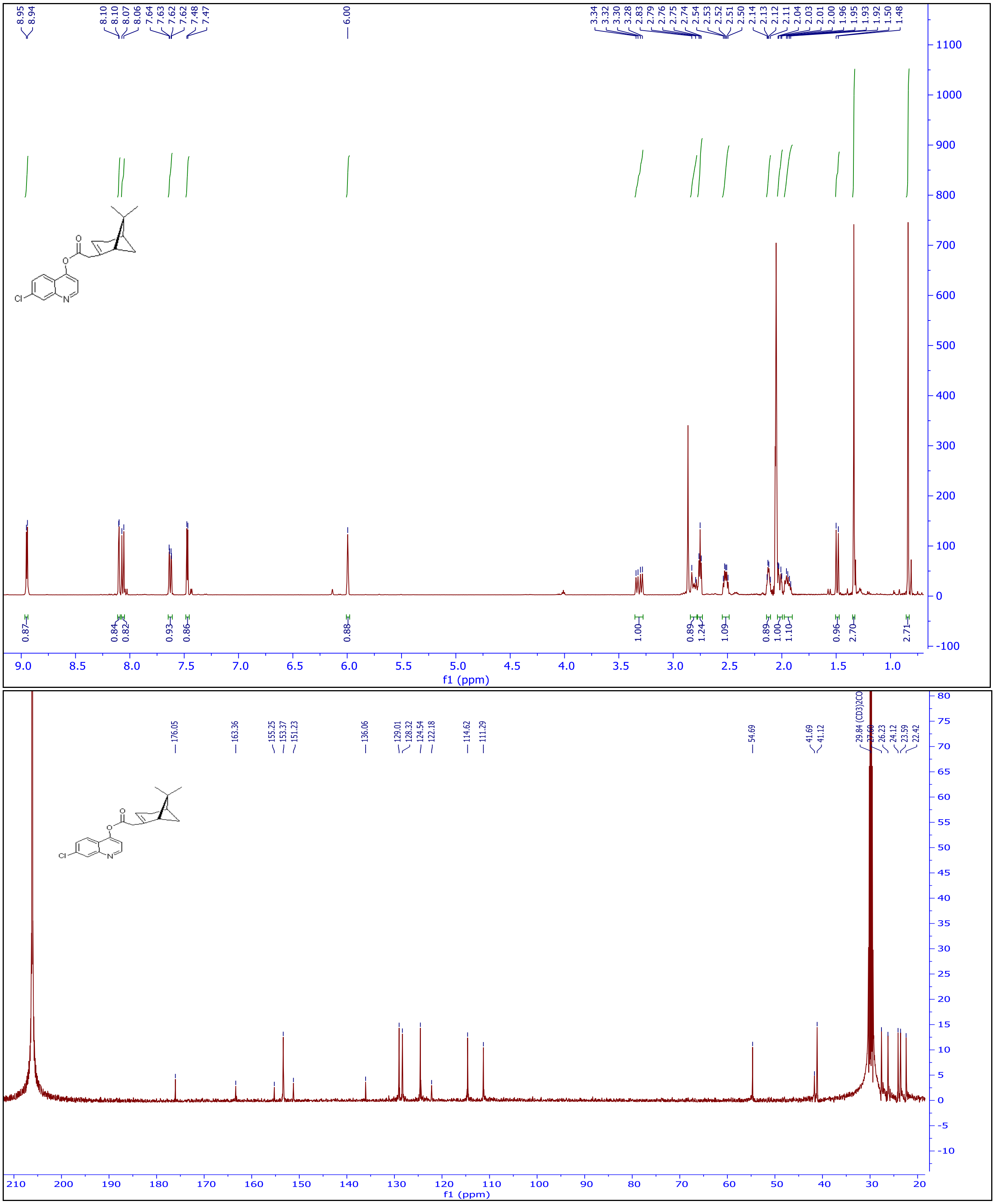

Figure 1.8a: (Top) ^{1}H-NMR and (Bottom) ^{13}C-NMR of 7-chloroquinolin-4-yl 2-((1*S*,5*R*)-6,6-dimethylbicyclo [3.1.1] hept-2-en-2-yl) acetate (**8**) in $(CD_3)_2CO$.

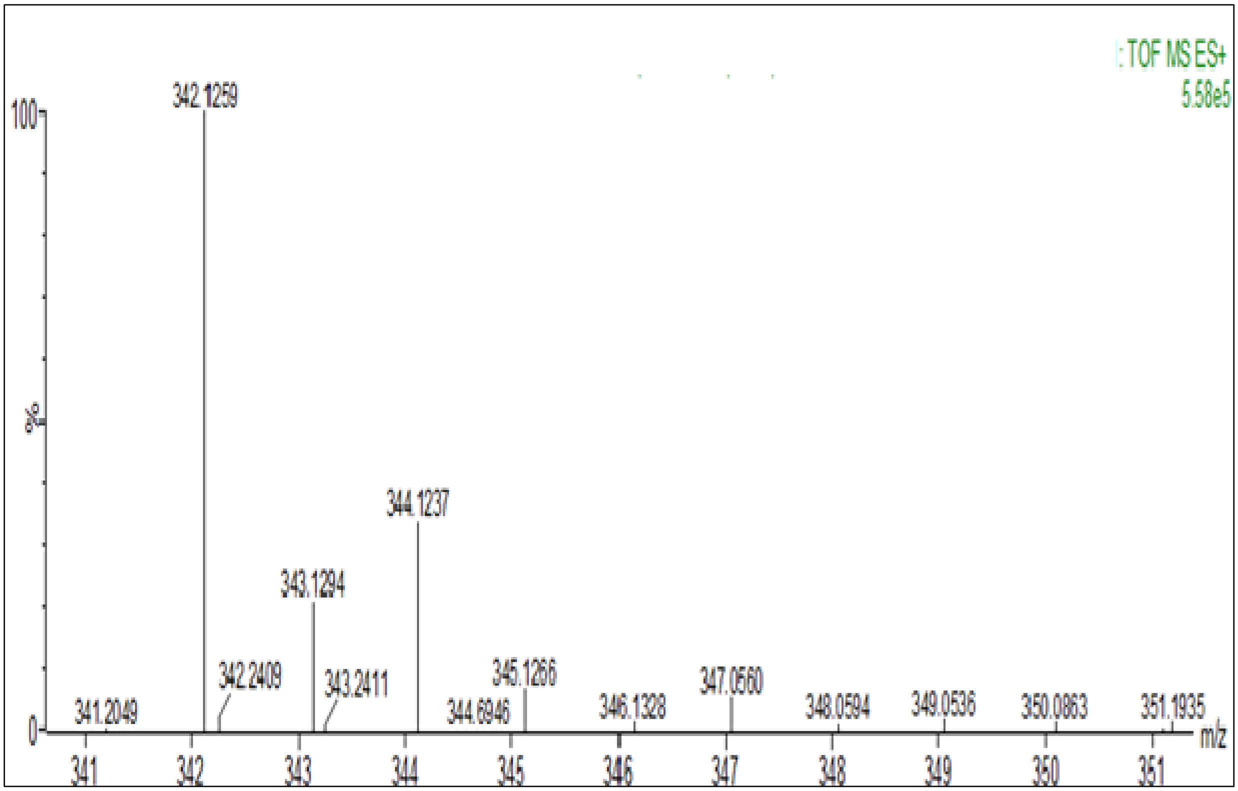

Figure 1.8b: HRMS $[M+H]^+$: 342.1259 m/z for 7-chloroquinolin-4-yl 2-((1*S*,5*R*)-6,6-dimethylbicyclo [3.1.1] hept-2-en-2-yl) acetate (**8**).

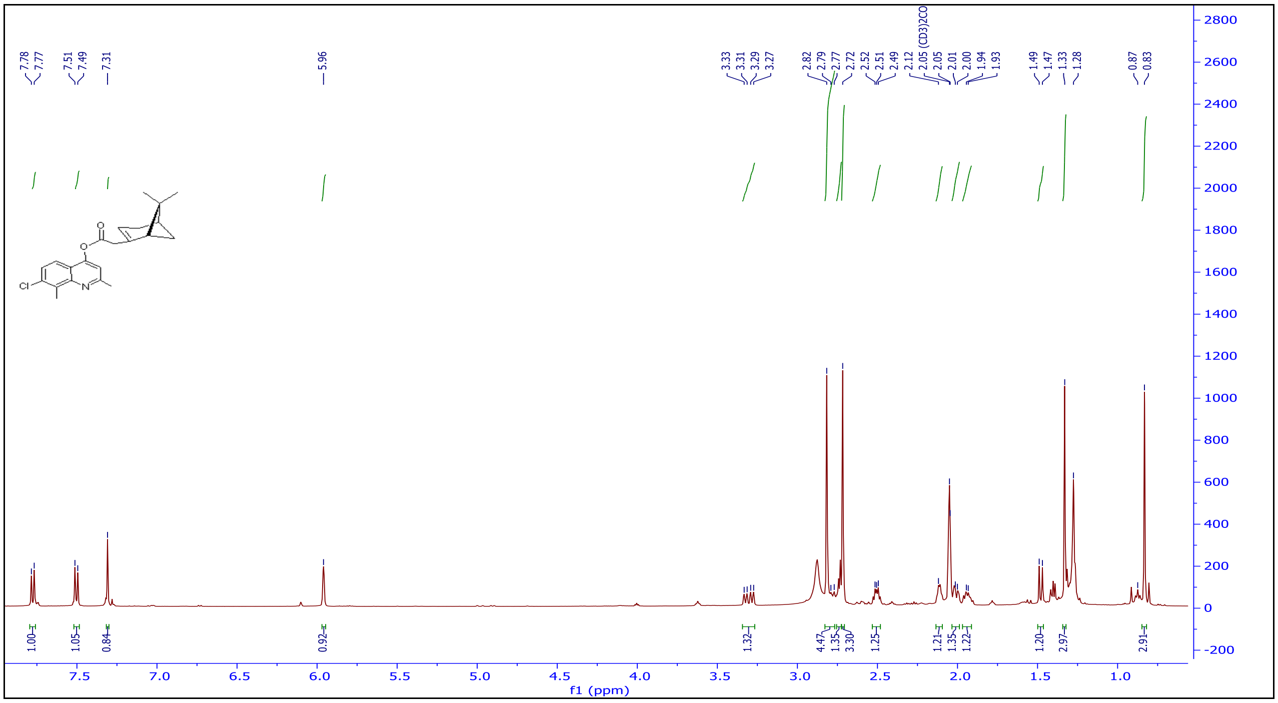

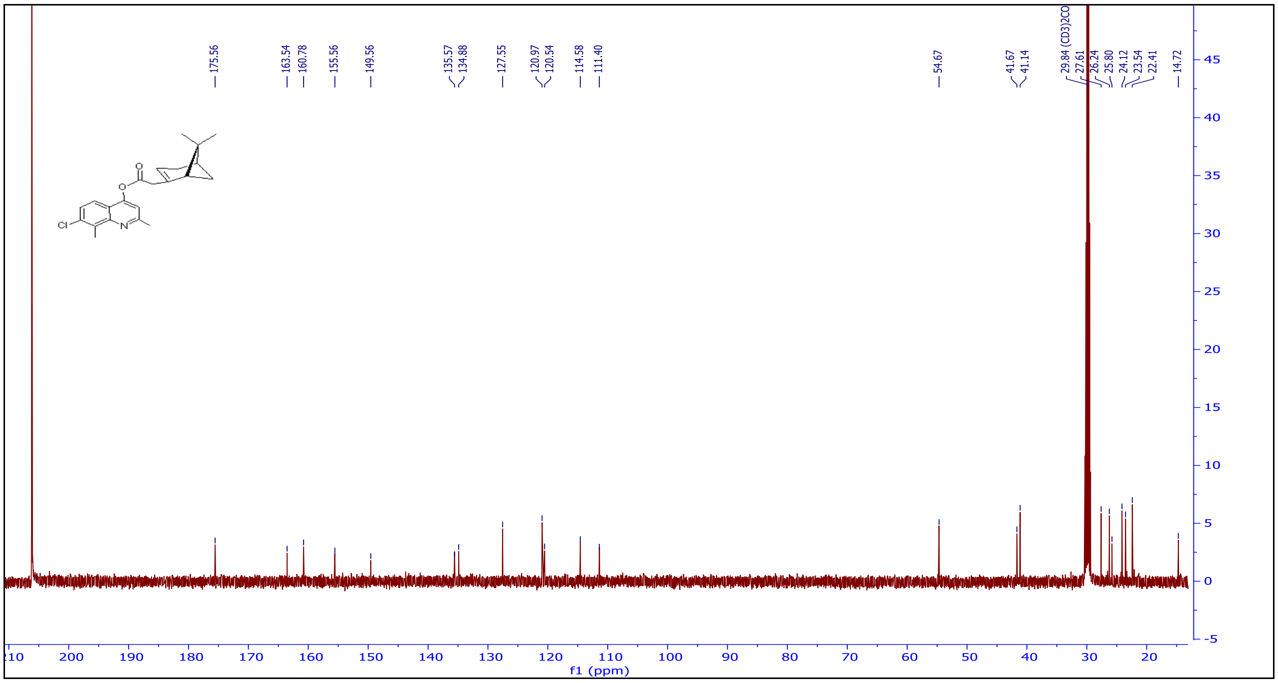

Figure 1.9a: (Top) ^{1}H-NMR and (Bottom) ^{13}C-NMR of 7-chloro-2,8-dimethylquinolin-4-yl 2-((1*S*,5*R*)-6,6-dimethylbicyclo [3.1.1] hept-2-en-2-yl) acetate (**9**) in $(CD_3)_2CO$.

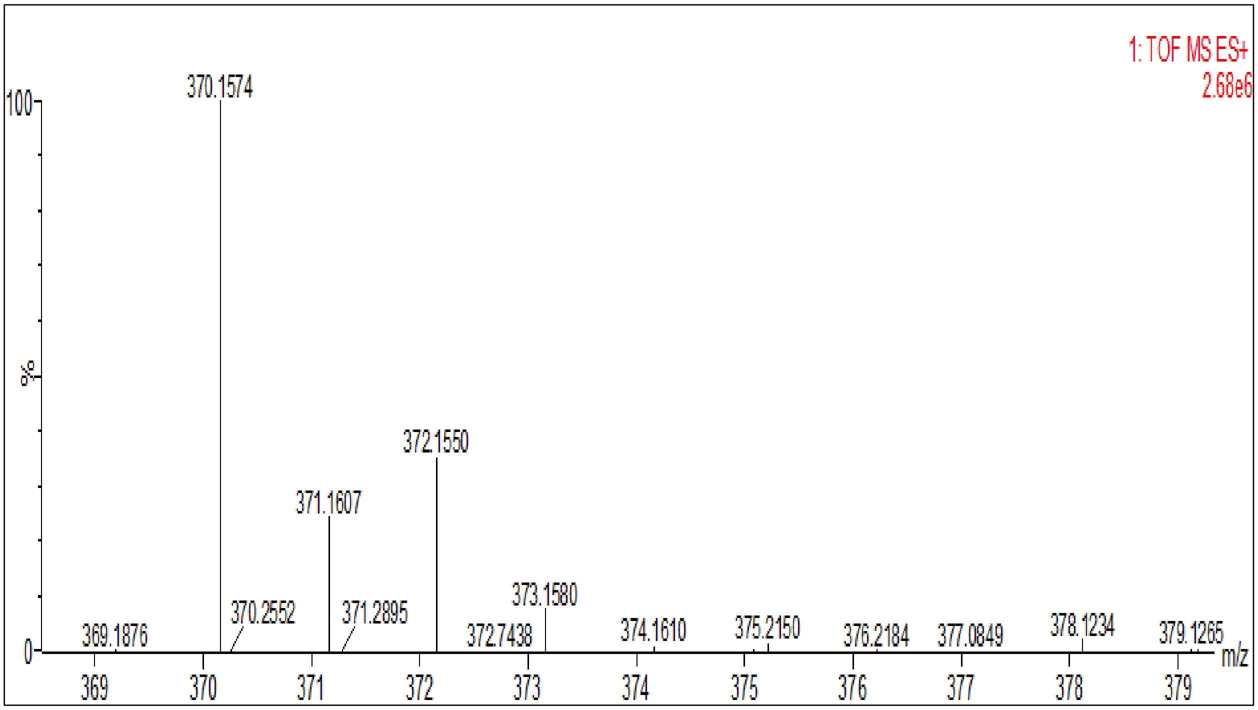

Figure 1.9b: HRMS: $[M+H]^+$: 370.1574 m/z for 7-chloro-2,8-dimethylquinolin-4-yl 2-((1S,5R)-6,6-dimethylbicyclo [3.1.1] hept-2-en-2-yl) acetate (**9**).

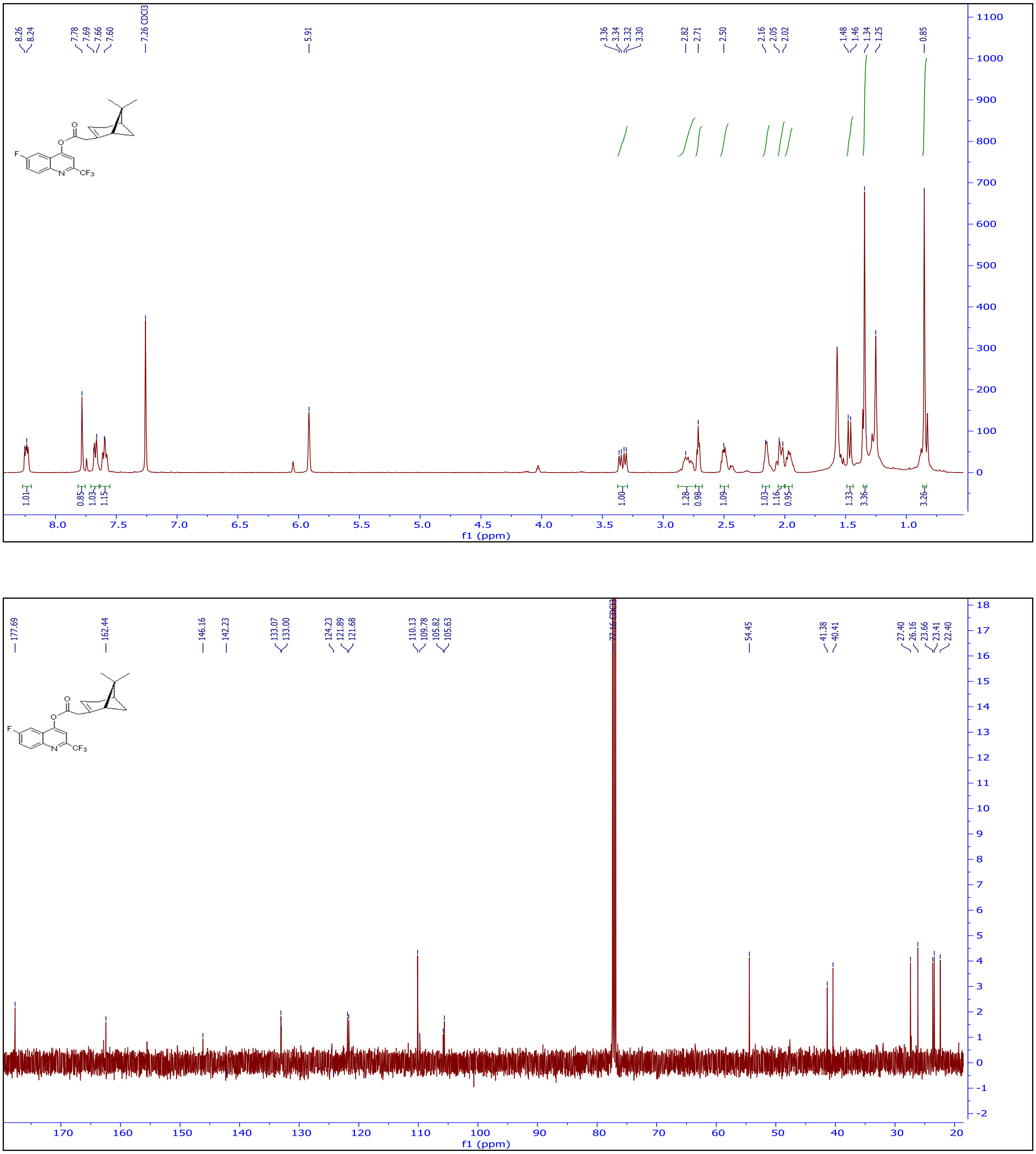

Figure 1.10a: (Top) ^{1}H-NMR and (Bottom) ^{13}C-NMR of 6-fluoro-2-(trifluoromethyl) quinolin-4-yl 2-((1*S*,5*R*)-6,6-dimethylbicyclo [3.1.1] hept-2-en-2-yl) acetate (**10**) in $CDCl_3$.

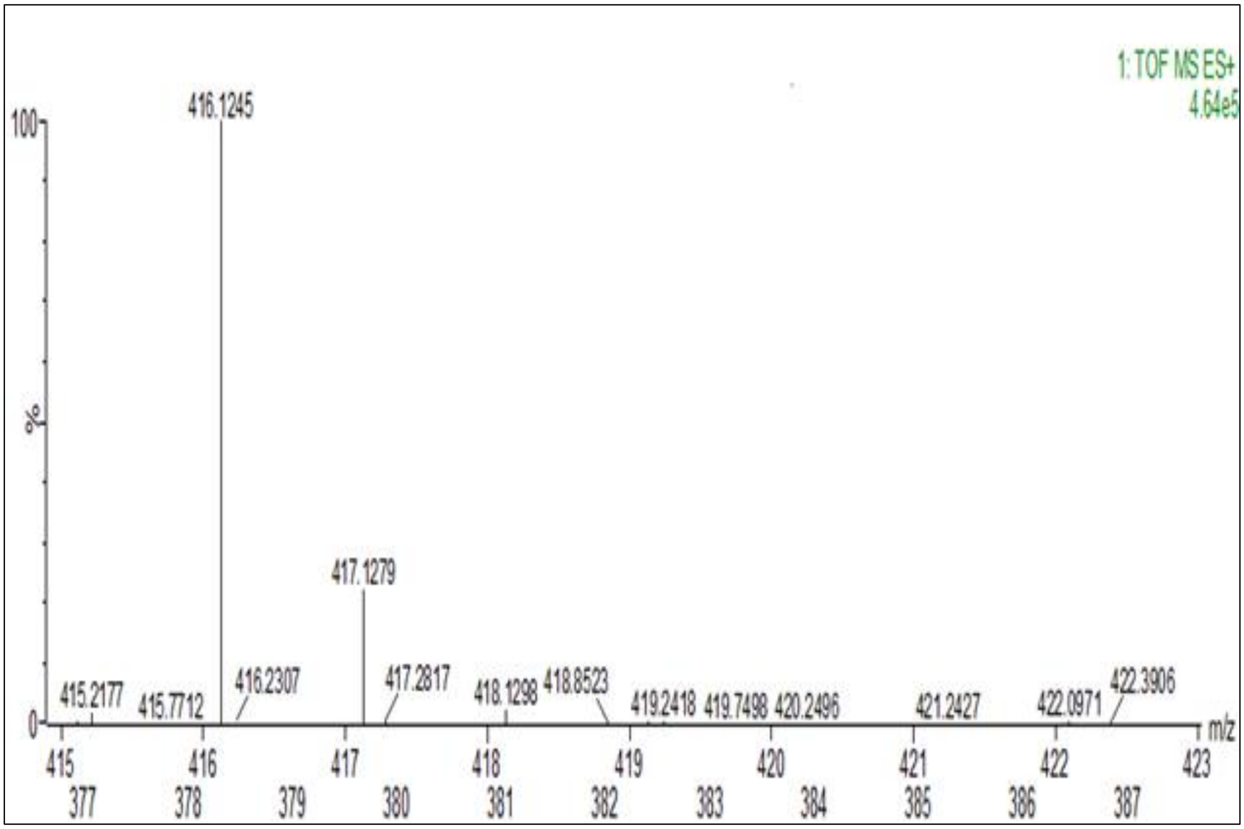

Figure 1.10b: HRMS: $[M+Na]^+$: 416.1245 m/z for 6-fluoro-2-(trifluoromethyl) quinolin-4-yl 2-((1*S*,5*R*)-6,6-dimethylbicyclo [3.1.1] hept-2-en-2yl) (**10**).

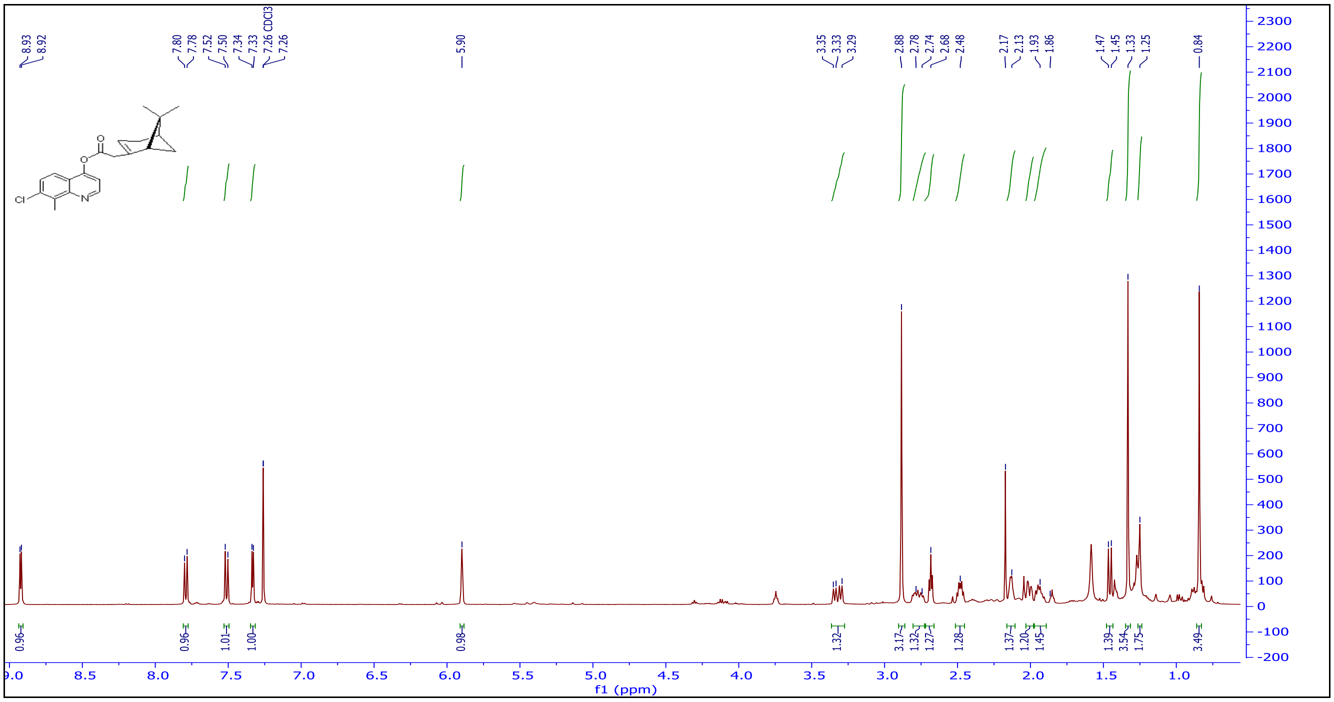

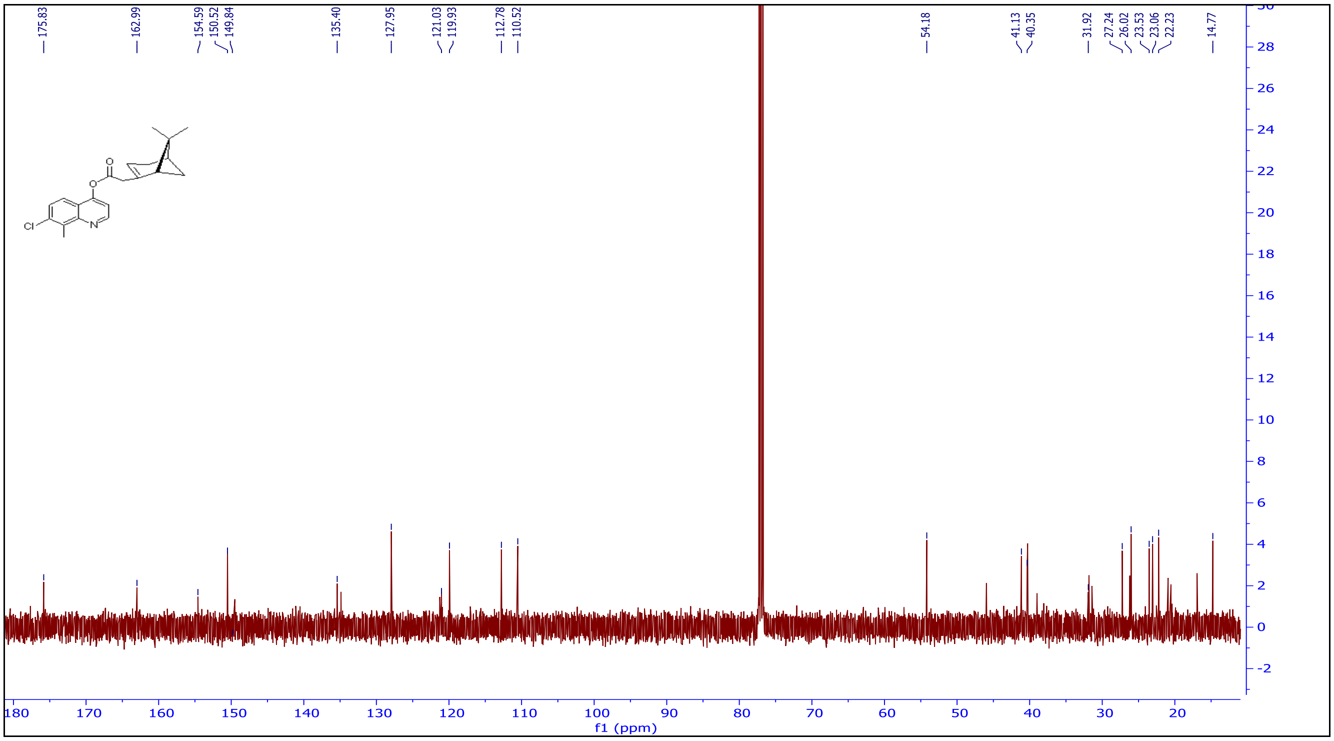

Figure 1.11: (Top) ^{1}H-NMR and (Bottom) ^{13}C-NMR of 7-chloro-8-methylquinolin-4-yl 2-((1*S*,5*R*)-6,6-dimethylbicyclo [3.1.1] hept-2-en-2-yl) acetate (**11**) in $CDCl_3$.

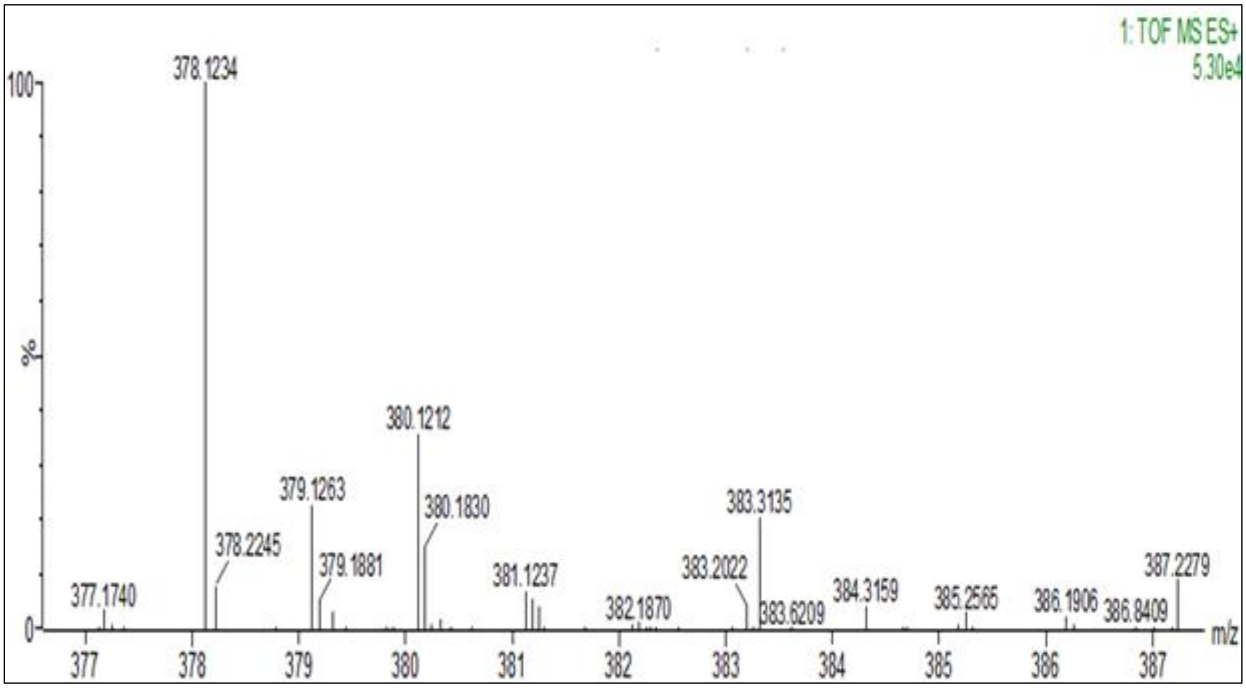

Figure 1.11b: HRMS: [M+Na]$^+$: 378.1234 m/z for 7-chloro-8-methylquinolin-4-yl 2-((1*S*,5*R*)-6,6-dimethylbicyclo [3.1.1] hept-2-en-2-yl) acetate (**11**).

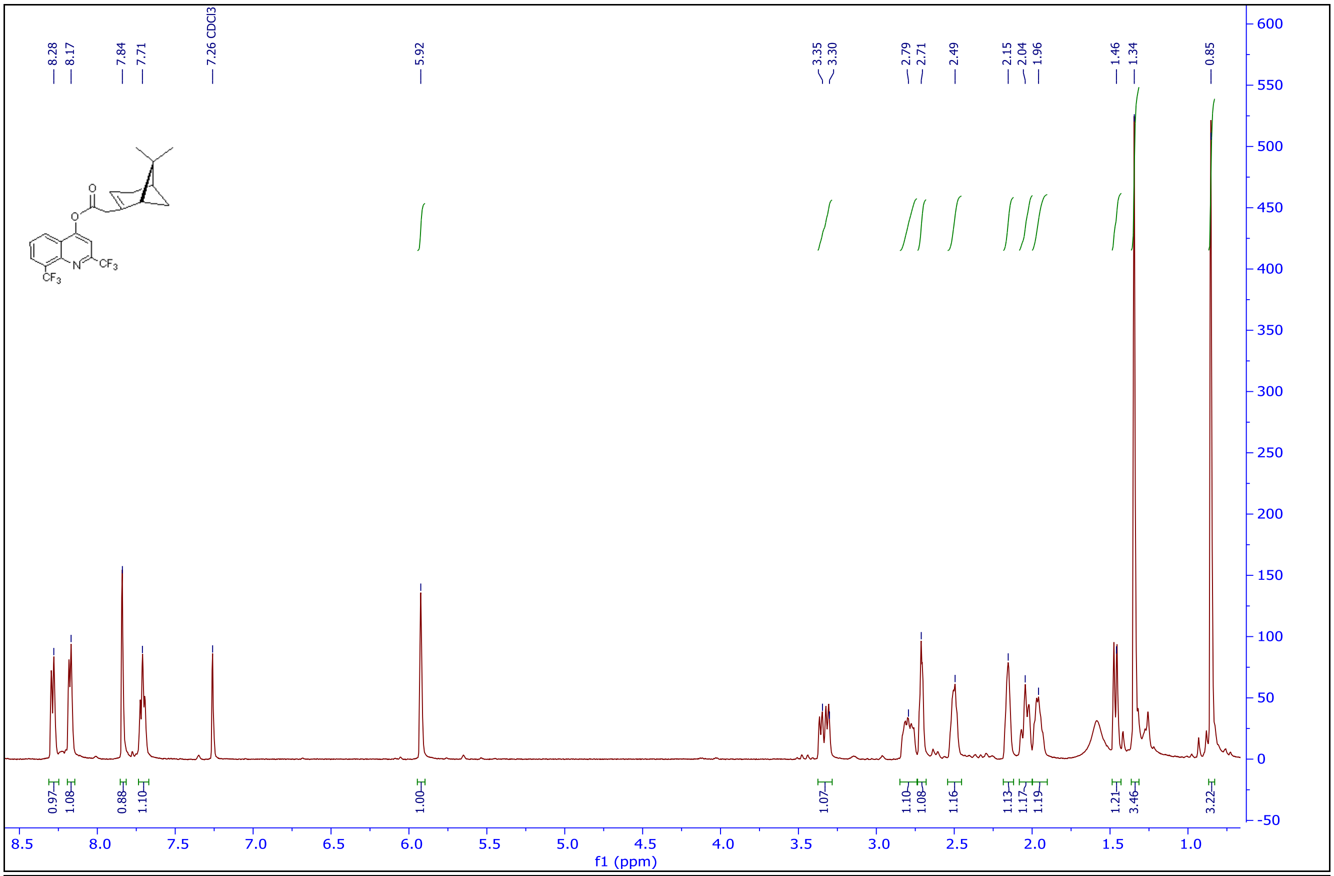

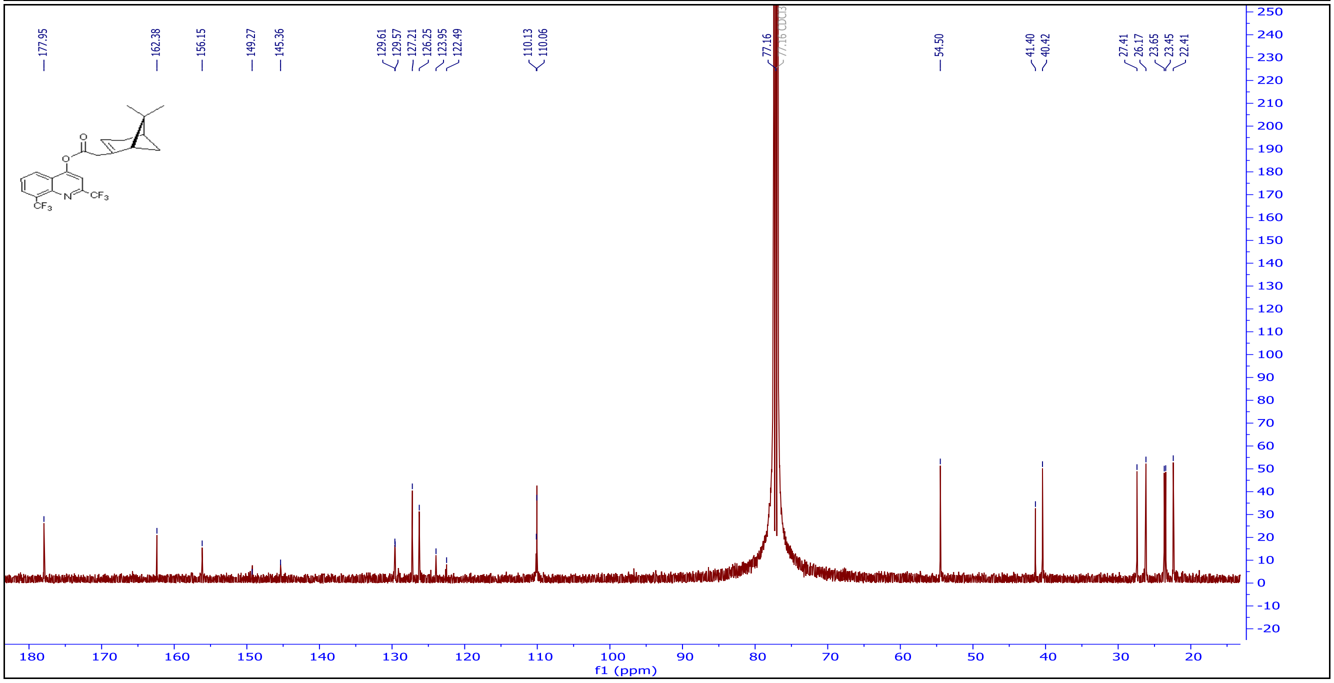

Figure 1.12a: (Top)^{1}H-NMR and (Bottom) ^{13}C-NMR of 2,8 bis (trifluoromethyl)quinolin-4-yl 2-((1*S*,5*R*)-6,6-dimethylbicyclo [3.1.1] hept-2-en-2-yl) acetate (**12**) in $CDCl_3$.

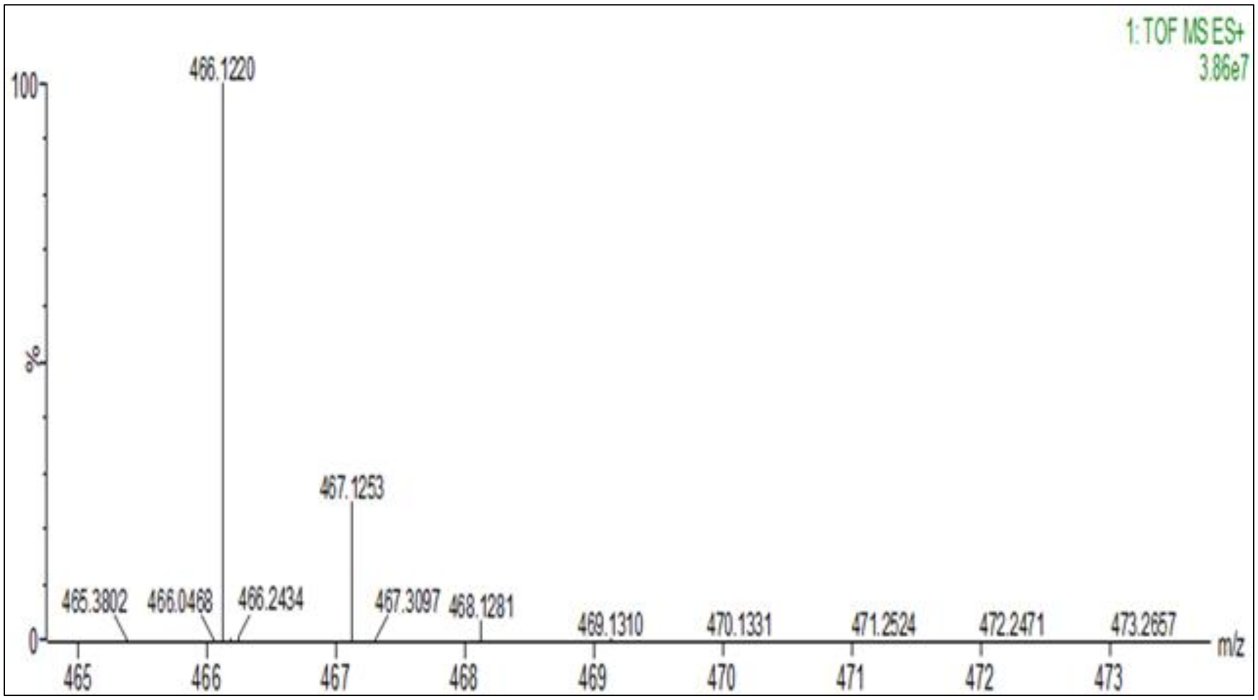

Figure 1.12b: HRMS: $[M+Na]^+$: 466.1220 m/z for 8-bis(trifluoromethyl)quinolin-4-yl 2-((1*S*,5*R*)-6,6-dimethylbicyclo [3.1.1] hept-2-en-2-yl) acetate (**12**).

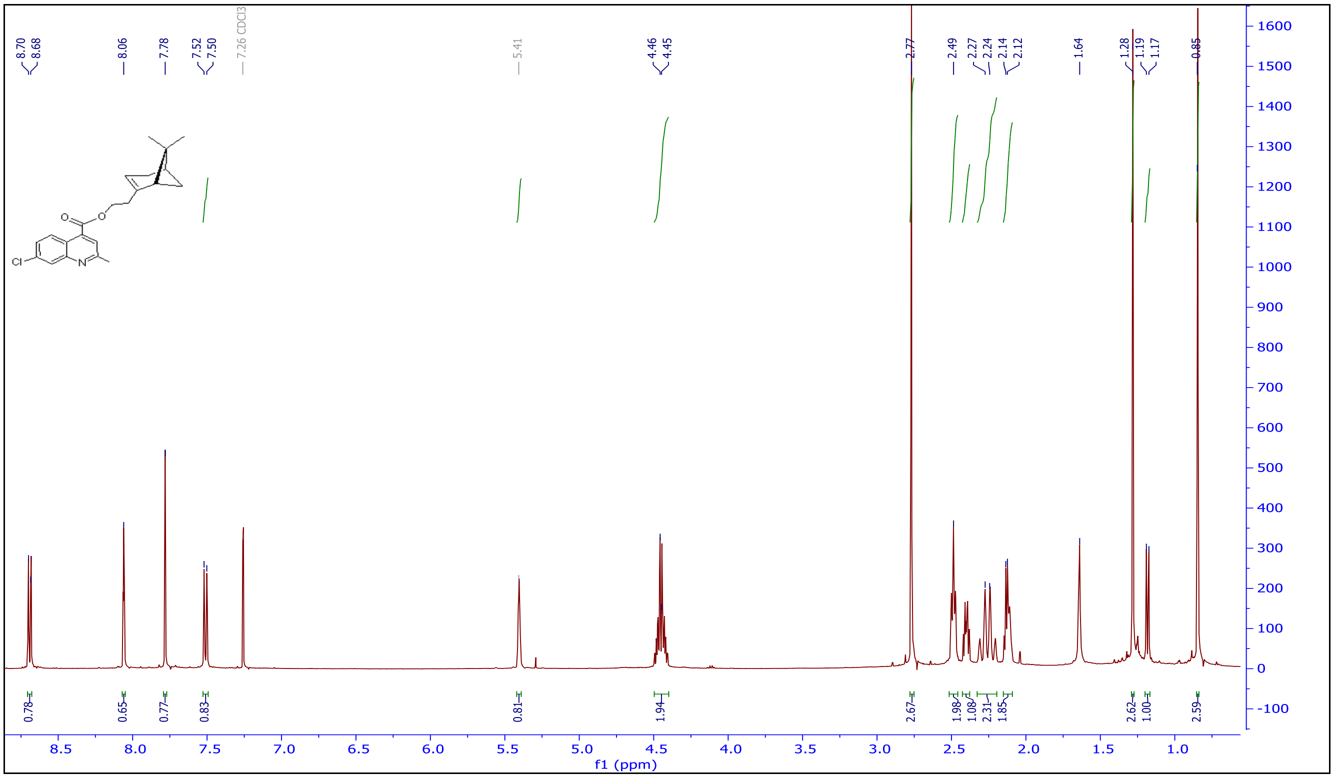

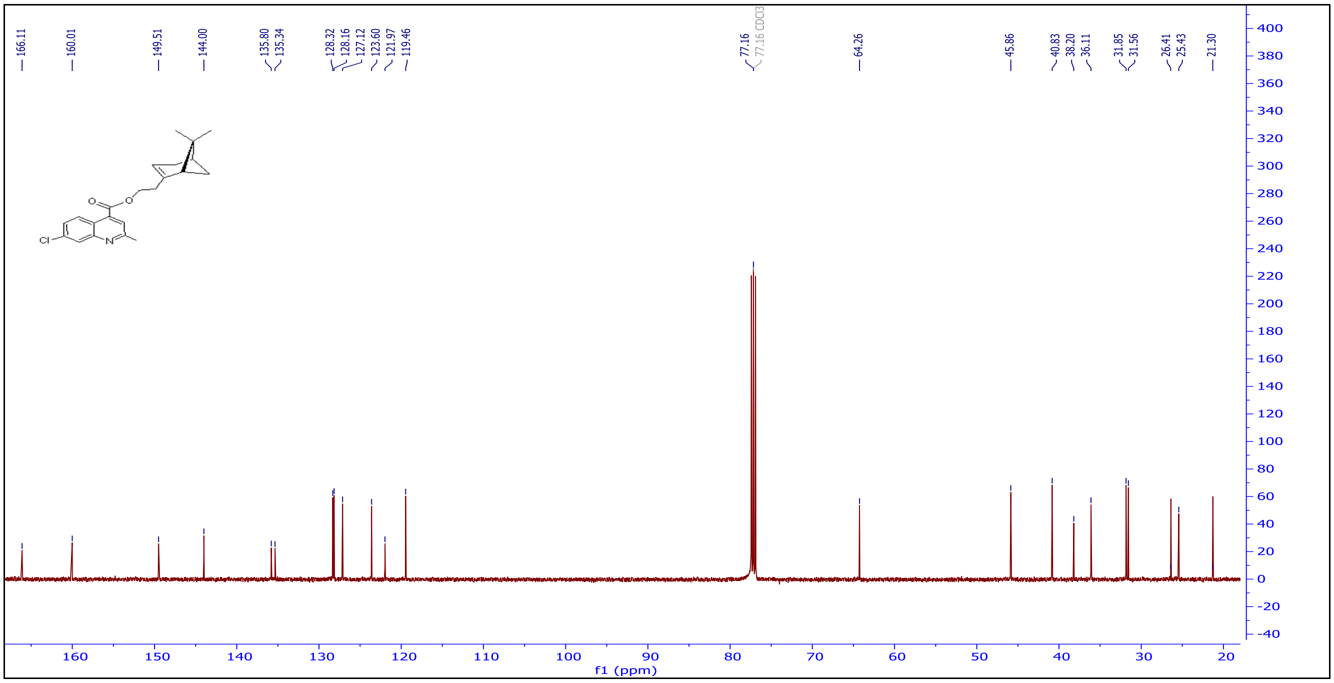

Figure 1.13a: (Top) ^{1}H-NMR and (Bottom) ^{13}C-NMR of 2-((1*S*,5*R*)-6,6-dimethylbicyclo [3.1.1] hept-2-en-2-yl) ethyl 7-chloro-2-methylquinoline-4-carboxylate (**13**) in $CDCl_3$.

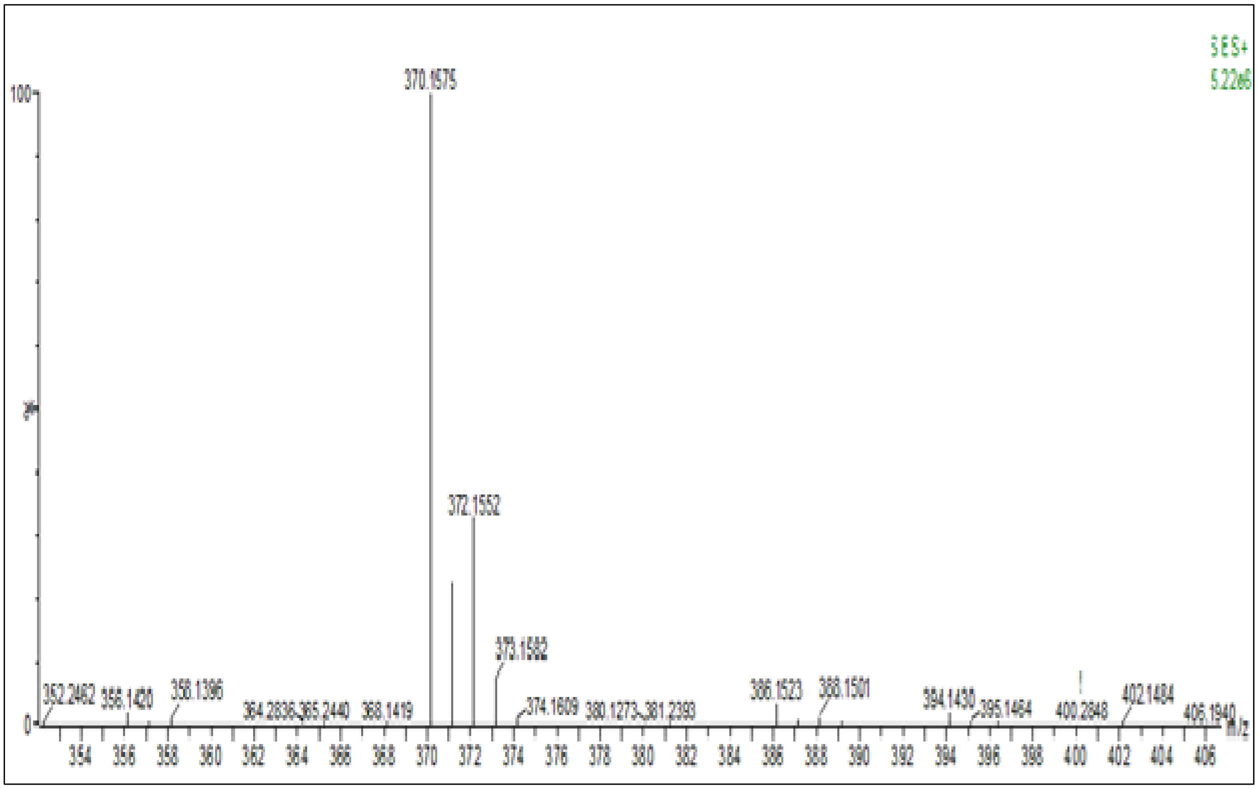

Figure 1.13b: HRMS: $[M+H]^+$: 370.1575 m/z for 2-((1*S*,5*R*)-6,6-dimethylbicyclo [3.1.1] hept-2-en-2-yl) ethyl 7-chloro-2-methylquinoline-4-carboxylate (**13**).

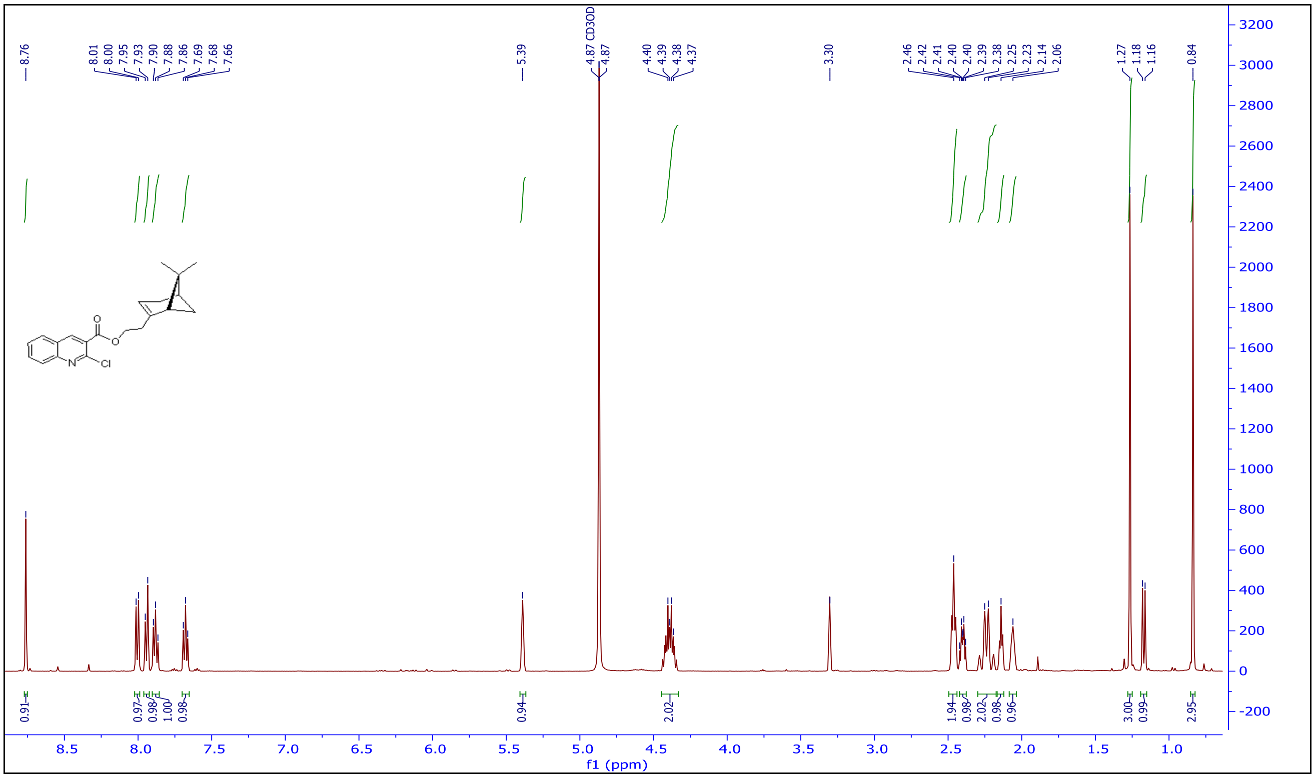

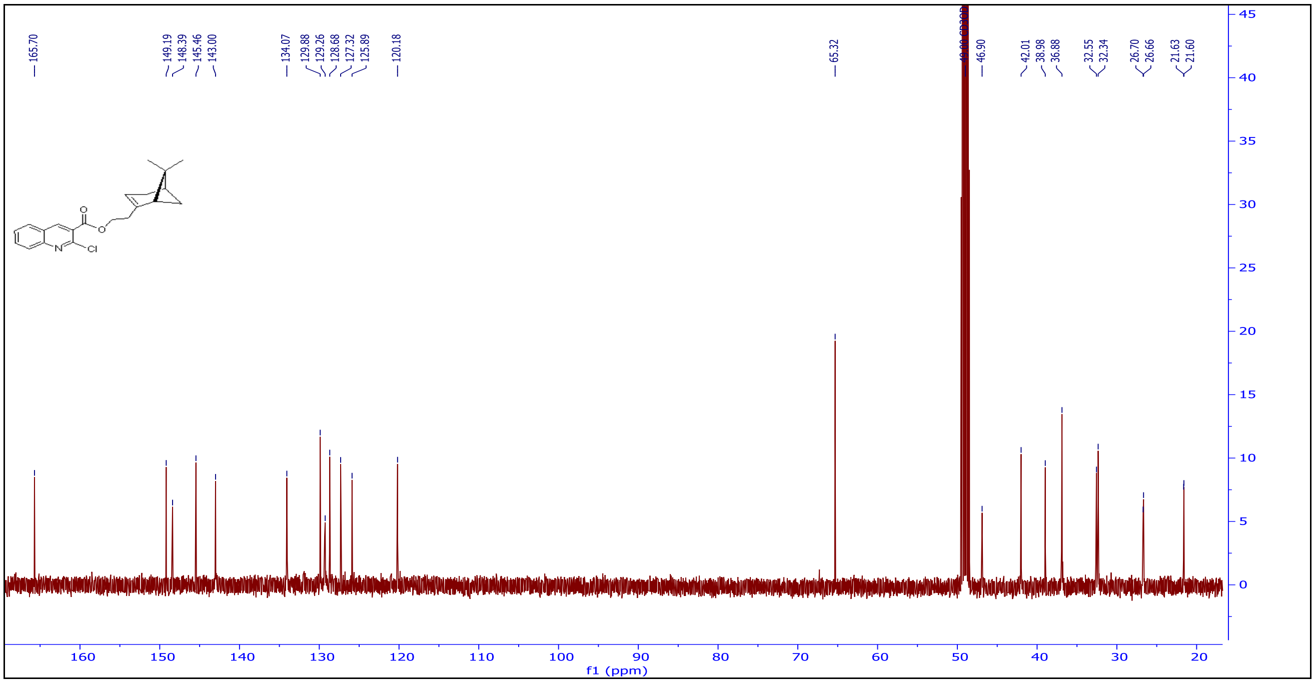

Figure1.14a: (Top) ^{1}H-NMR and (Bottom) ^{13}C-NMR of 2-((1S,5R)-6,6-dimethylbicyclo [3.1.1] hept-2-en-2-yl) ethyl 2-chloroquinoline-3-carboxylate (**14**) in CD_3OD.

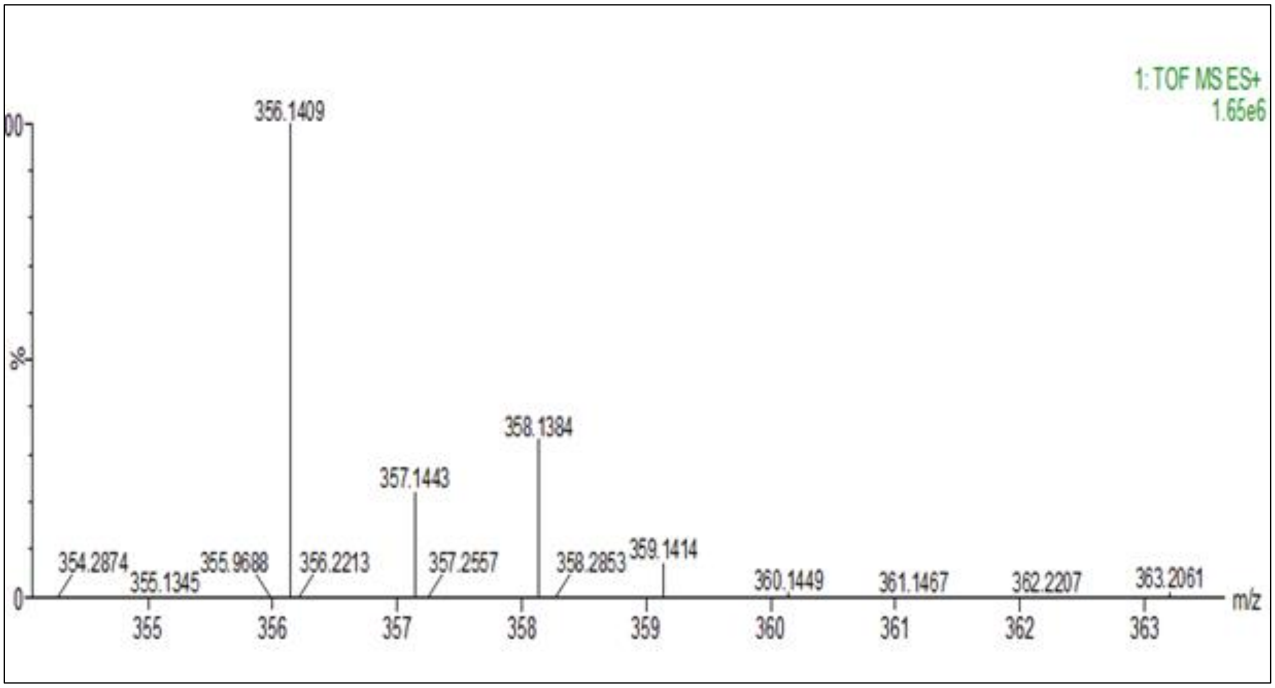

Figure 1.14b: HRMS: $[M+H]^+$: 356.1417 m/z for 2-((1*R*, 5*S*)-6, 6-dimethylbicyclo [3.1.1] hept-2-en-2-yl) ethyl 2-chloroquinoline-3-carboxylate (**14**).

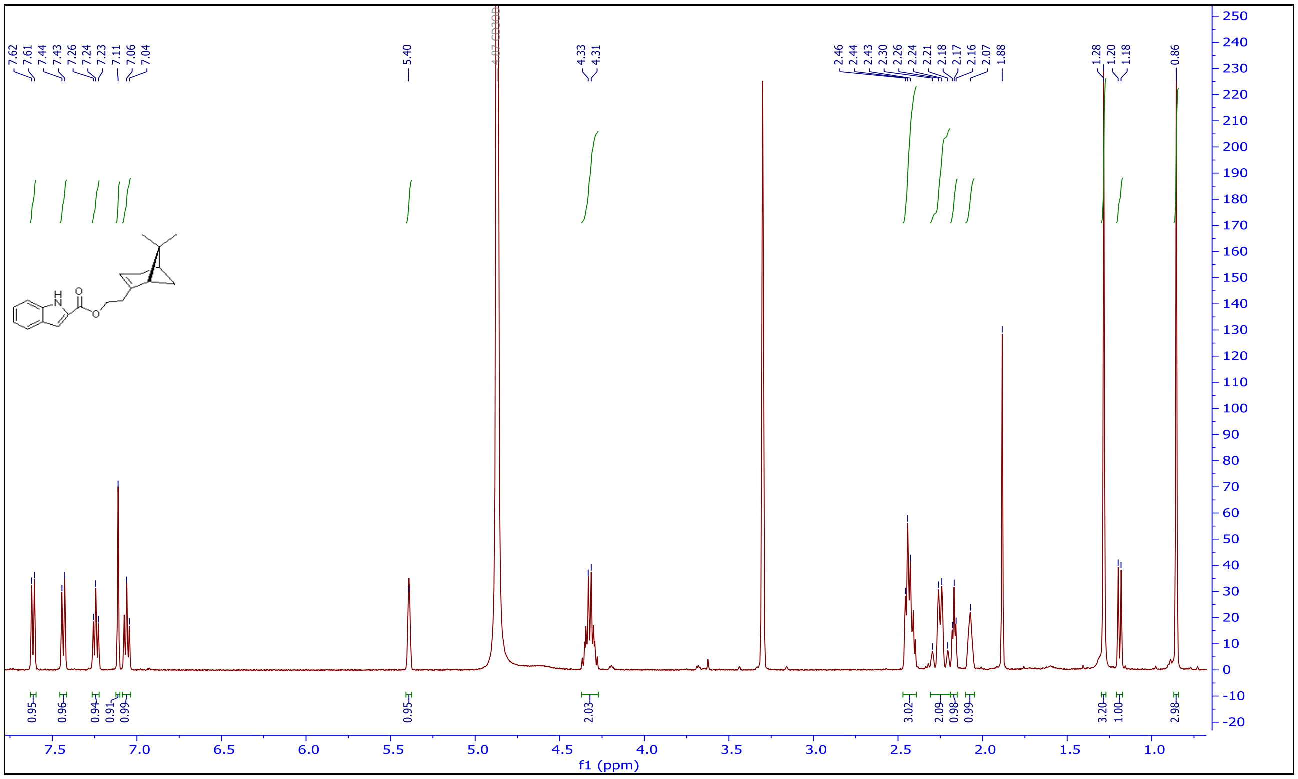

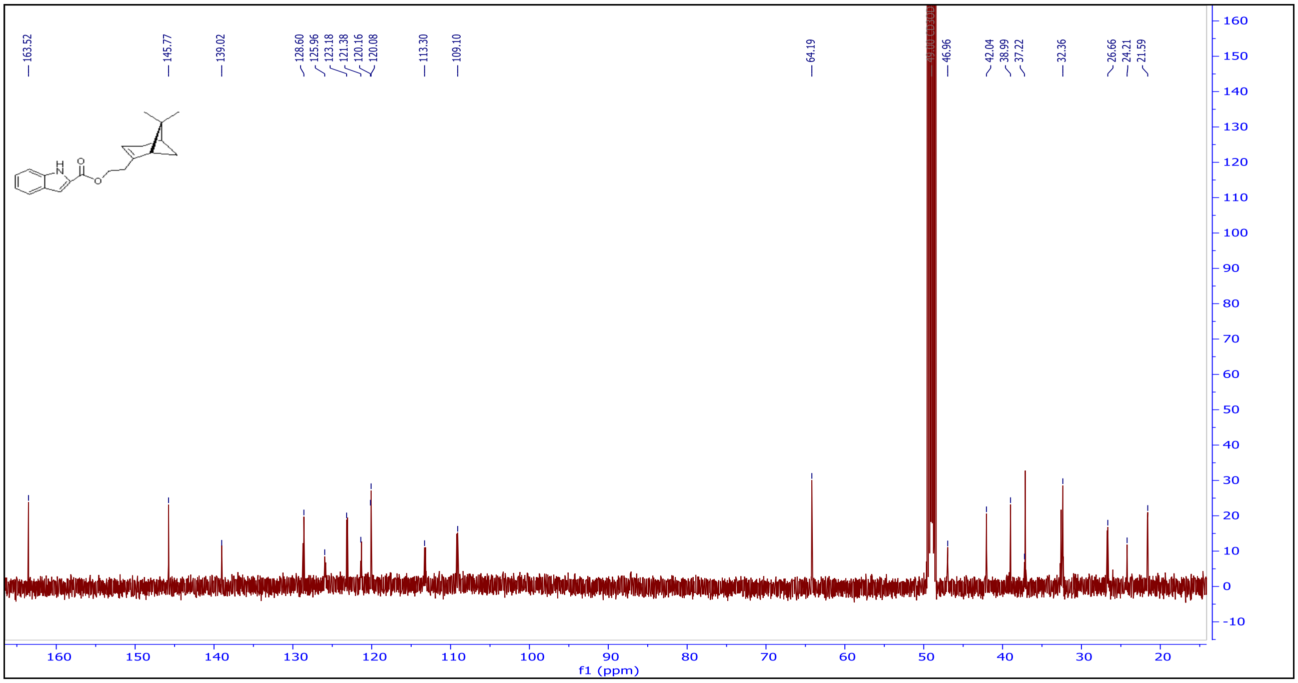

Figure 1.15a: (Top) ^{1}H-NMR and (Bottom) ^{13}C-NMR of 2-((1*R*,5*S*)-6,6-dimethylbicyclo [3.1.1] hept-2-en-2-yl) ethyl 1*H*-indole-2-carboxylate (**15**) in MeOD.

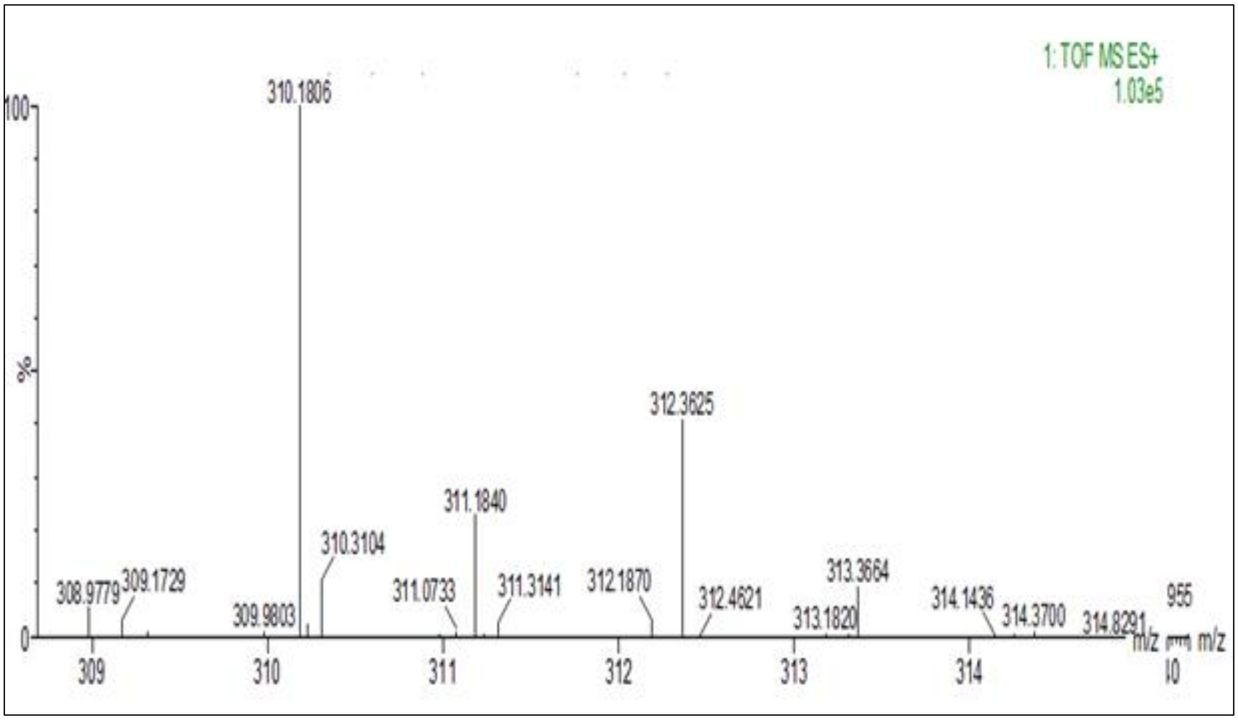

Figure 1.15b: HRMS: $[M+H]^+$: 310.1806 m/z for 2-((1*R*, 5*S*)-6, 6-dimethylbicyclo [3.1.1] hept-2-en-2-yl) ethyl 1H-indole-2-carboxylate (**15**).

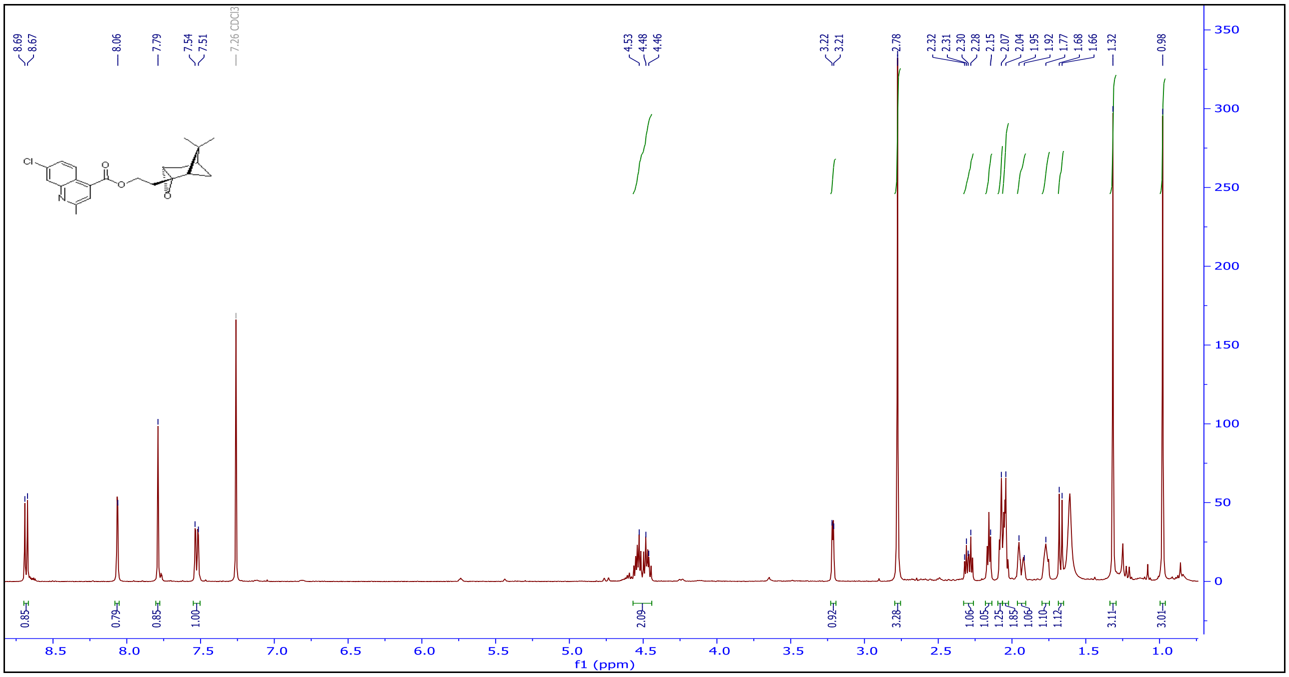

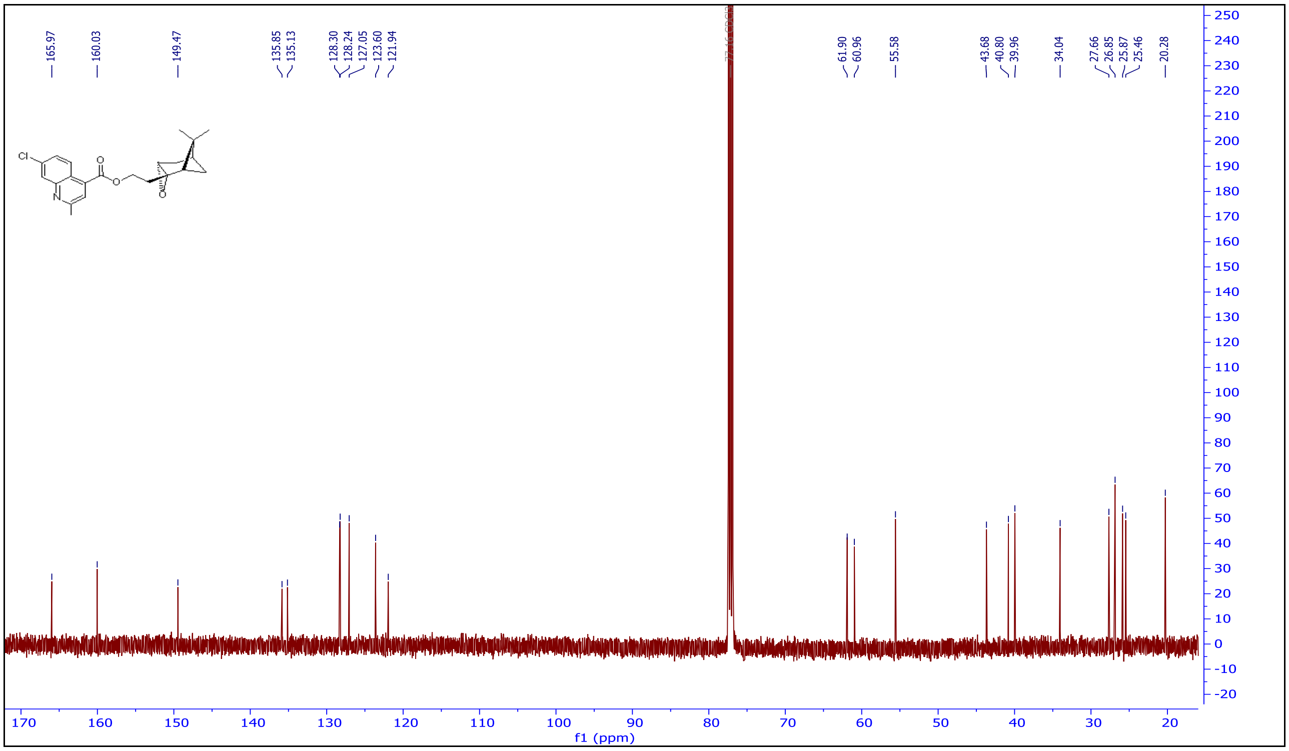

Figure 1.16a: (Top) ^{1}H-NMR and (Bottom) ^{13}C-NMR of 2-((1*S*,2*S*,4*R*,6*S*)-7,7-dimethyl-3-oxatricyclo [4.1.1.0^{2,4}] octan-2-yl) ethyl-7-chloro-2 methylquinoline-4-carboxylate (**16**) in $CDCl_3$.

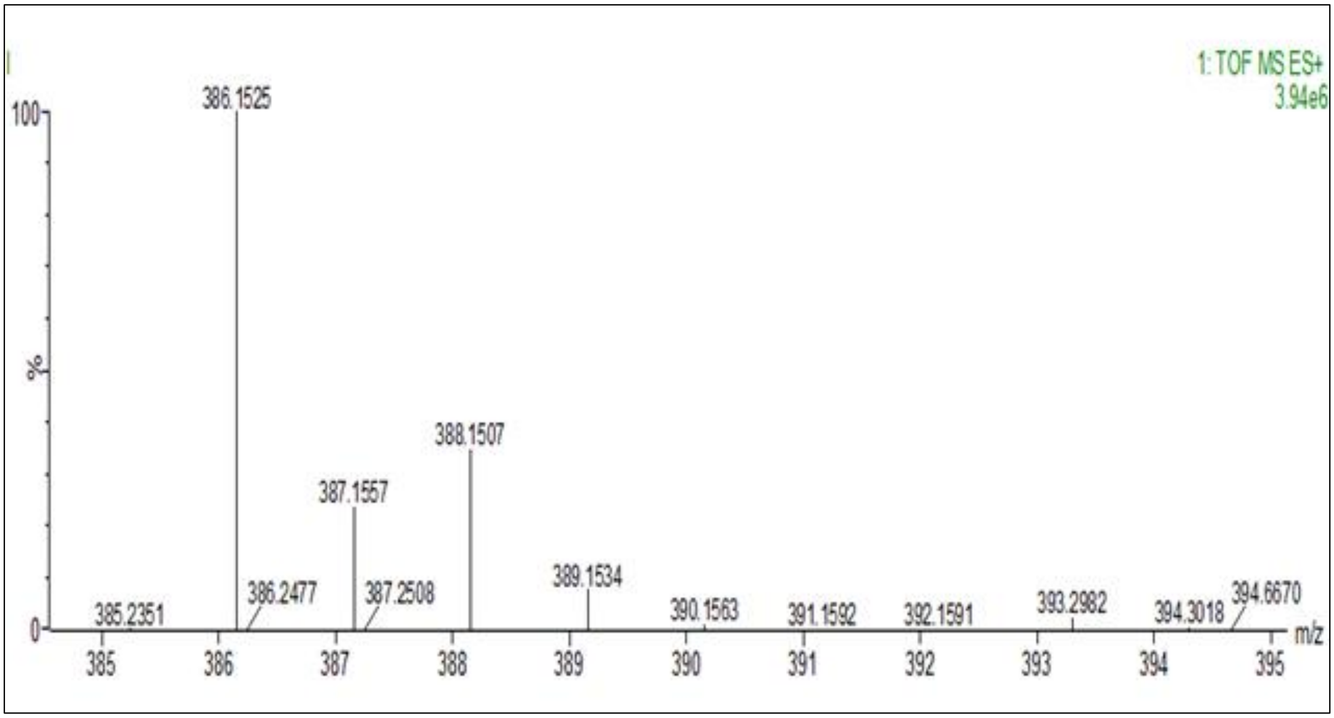

Figure 1.16b: HRMS: $[M+H]^+$: 386.1525 m/z for 2-((1*S*,2*S*,4*R*,6*S*)-7,7-dimethyl-3-oxatricyclo [4.1.1.0^{2,4}] octan-2-yl) ethyl 7-chloro-2-methylquinoline-4-carboxylate (**16**).
